# Transcriptomics- and 3D imaging–based characterization of the lymphatic vasculature in human skin

**DOI:** 10.1084/jem.20242353

**Published:** 2025-11-04

**Authors:** Aline Bauer, Lito Zambounis, Ioannis Kritikos, Almut Lütge, Amélie Sabine, Coraline Heron, Milena Petkova, Costanza Giampietro, Katharina Blatter, Salvatore Daniele Bianco, Daniel Gschwend, Gaetana Restivo, Steven T. Proulx, Mitchell P. Levesque, Nicole Lindenblatt, Edoardo Mazza, Michael Detmar, Epameinondas Gousopoulos, Mark D. Robinson, Tatiana V. Petrova, Cornelia Halin

**Affiliations:** 1 Institute of Pharmaceutical Sciences, ETH Zurich, Zurich, Switzerland; 2 Department of Molecular Life Sciences and SIB Swiss Institute of Bioinformatics, University of Zurich, Zurich, Switzerland; 3 Department of Fundamental Oncology, CHUV and University of Lausanne, Epalinges, Switzerland; 4 Swiss Federal Laboratories for Materials Science and Technology, Dübendorf, Switzerland; 5 Department of Mechanical and Process Engineering, Institute for Mechanical Systems, ETH Zurich, Zurich, Switzerland; 6 Bioinformatics Laboratory, Fondazione IRCCS Casa Sollievo della Sofferenza, San Giovanni Rotondo (FG), Italy; 7 Department of Dermatology, University Hospital Zurich and University of Zurich, Schlieren, Switzerland; 8 Theodor Kocher Institute, University of Bern, Bern, Switzerland; 9 Department of Plastic Surgery and Hand Surgery, University Hospital Zurich, Zurich, Switzerland

## Abstract

Afferent lymphatic vessels (LVs) are present in most vascularized tissues and exert important immune and drainage functions, yet human afferent LVs remain poorly studied. Performing single-cell RNA sequencing of lymphatic endothelial cells (LECs) from human skin and subcutaneous adipose tissue, we identified various LEC subsets, including two valve LEC populations located on the upstream and downstream sides of the valve leaflets. The cell adhesion molecule CD24 emerged as a specific marker of upper valve leaflet LECs in human skin and contributed to lymphatic valve development in murine mesentery. Three-dimensional imaging further revealed several unique features of the human dermal lymphatic network, including a high proportion of LYVE-1^+^ pre-collecting vessels containing intraluminal valves, virtually no collectors, and absence of lymphatic muscle cell coverage. Moreover, LECs in blind-ended capillaries and around valves in pre-collectors displayed mixed junctional and morphological phenotypes. These findings reveal key differences between human and murine dermal afferent lymphatics and provide a deeper understanding of human lymphatic-related (patho)physiological processes.

## Teaser

This study reveals how the structure and cellular morphology of human dermal afferent lymphatic vessels differ from the current, primarily mouse tissue–based textbook description. Furthermore, it identifies the new valve marker CD24 and reveals its contribution to mesenteric valve formation.

## Introduction

Lymphatic vessels (LVs) are present in most vascularized tissues of the body and can be divided into three vascular compartments: the afferent LVs upstream of lymph nodes (LNs), the lymphatic network within LNs, and the efferent LVs downstream of LNs (Petrova and Koh, 2018). A key function of afferent LVs is to drain excess tissue fluid that has leaked out of blood vessels (BVs) and to return it to the blood circulation. At the same time, afferent LVs transport antigens and leukocytes from peripheral tissues to draining LNs (dLNs), where adaptive immune responses are initiated and regulated (Bauer et al., 2022; Oliver et al., 2020). Over the past six decades, seminal imaging-based studies have contributed to shaping the current view of the organization of the lymphatic network in peripheral tissues, such as in the murine skin. According to this model, afferent LVs start as blind-ended lymphatic capillaries, which are lined by oak leaf–shaped lymphatic endothelial cells (LECs) that are loosely interconnected through discontinuous button-like junctions. This setup gives rise to open flaps, also referred to as primary valves, that facilitate the uptake of interstitial fluid, macromolecules, and leukocytes into the capillary lumen (Baluk et al., 2007; Pflicke and Sixt, 2009). Anchoring filaments are fibers, which were observed as early as the 1960s in tissues from various mammalian species (e.g., guinea pig, rat, dog, mouse, and human) using scanning electron microscopy (Casley-Smith, 1967; Casley-Smith, 1980; Gerli et al., 1990; Leak and Burke 1968). These filaments are believed to connect the abluminal sides of LECs to the surrounding extracellular matrix (ECM) and to help maintain capillaries and primary valves in an open state, in particular under conditions of elevated interstitial tension (e.g., during edema). However, their function remains a matter of current debate (Baluk and McDonald, 2022). While lymphatic capillaries are specialized in lymph uptake, lymphatic collectors, which begin downstream of the capillaries, are primarily important for lymph transport. Accordingly, collector LECs are connected by continuous, cell–cell junctions and are underlined with a thick basement membrane (Baluk et al., 2007; Pflicke and Sixt, 2009). Moreover, lymphatic collectors are surrounded by lymphatic muscle cells (LMCs) and contain intraluminal valves, which divide collecting vessels into distinct units termed lymphangions. During LMC-mediated contraction of a collecting vessel, the valves help to prevent lymph backflow and ensure unidirectional propulsion of lymph toward dLNs (Bazigou and Makinen, 2013; Moore and Bertram, 2018; Zawieja, 2009). In addition, recent reviews increasingly refer to segments known as pre-collector vessels. The latter are found between capillaries and collectors and share characteristic features of both vessel types; e.g., they display a mixed junctional phenotype, may contain valves, but have no or only sparse LMC coverage (Breslin et al., 2018; Petrova and Koh, 2020; Scallan and Jannaway, 2022; Ulvmar and Makinen, 2016).

Our current knowledge of lymphatic morphology, particularly of the junctional setup and composition of lymphatic capillaries and collectors, mainly derives from confocal- and multiphoton-based analyses of tissue whole mounts performed in the mouse trachea (Baluk et al., 2007) and various other murine tissues, such as in the ear skin, intestine, mesentery, or diaphragm (reviewed in Petrova and Koh [2018]). Moreover, single-cell RNA sequencing (scRNA-seq)–based approaches have recently shed light on the gene expression differences and the LEC subset division in murine afferent LVs (Gonzalez-Loyola et al., 2021; Petkova et al., 2023) or murine LNs (Fujimoto et al., 2020; Sibler et al., 2021). In contrast, the composition and organization of the lymphatic vasculature in human tissues is just starting to be characterized by three-dimensional (3D) imaging (Hagerling et al., 2017; Wang et al., 2014) or by scRNA-seq (Emont et al., 2022; He et al., 2022; Hong et al., 2023; Kumar et al., 2023; Li et al., 2021; Pan et al., 2024). A general hurdle when investigating endothelial cells (ECs) using scRNA-seq is that ECs only comprise a minor fraction of cells present in tissue, with LECs being even scarcer compared with blood vascular ECs (BECs). Two studies so far have provided a detailed description of the composition of human LEC subsets isolated by FACS from human LNs (Abe et al., 2022; Takeda et al., 2019). Conversely, scRNA-seq analyses performed on all cells isolated from a tissue typically failed to capture sufficient events and detailed LEC phenotypes. Consequently, the handful of studies that performed scRNA-seq on either human skin, breast, or adipose tissue only reported a few thousand LECs and placed little emphasis on their analysis (Emont et al., 2022; He et al., 2022; Kumar et al., 2023). Thus, at present, our understanding of distinct LEC subsets and their anatomical localization in human afferent LVs is still very limited.

In this study, we have combined 3D imaging and scRNA-seq to provide an in-depth characterization of the lymphatic vasculature in human skin and subcutaneous (s.c.) adipose tissue. Following this approach, we made several observations that reveal significant differences in organization of human and murine skin LVs, which can be relevant for a better understanding of human pathophysiological processes, such as lymphedema. Moreover, we identified the adhesion molecule CD24 as a novel, flow-induced marker of LECs present on the upstream sides of valve leaflets in human skin and demonstrated its contribution to mesenteric valve formation in mice.

## Results

### Analysis of the lymphatic network in human skin and adipose tissue by 3D imaging

**Figure 1.**
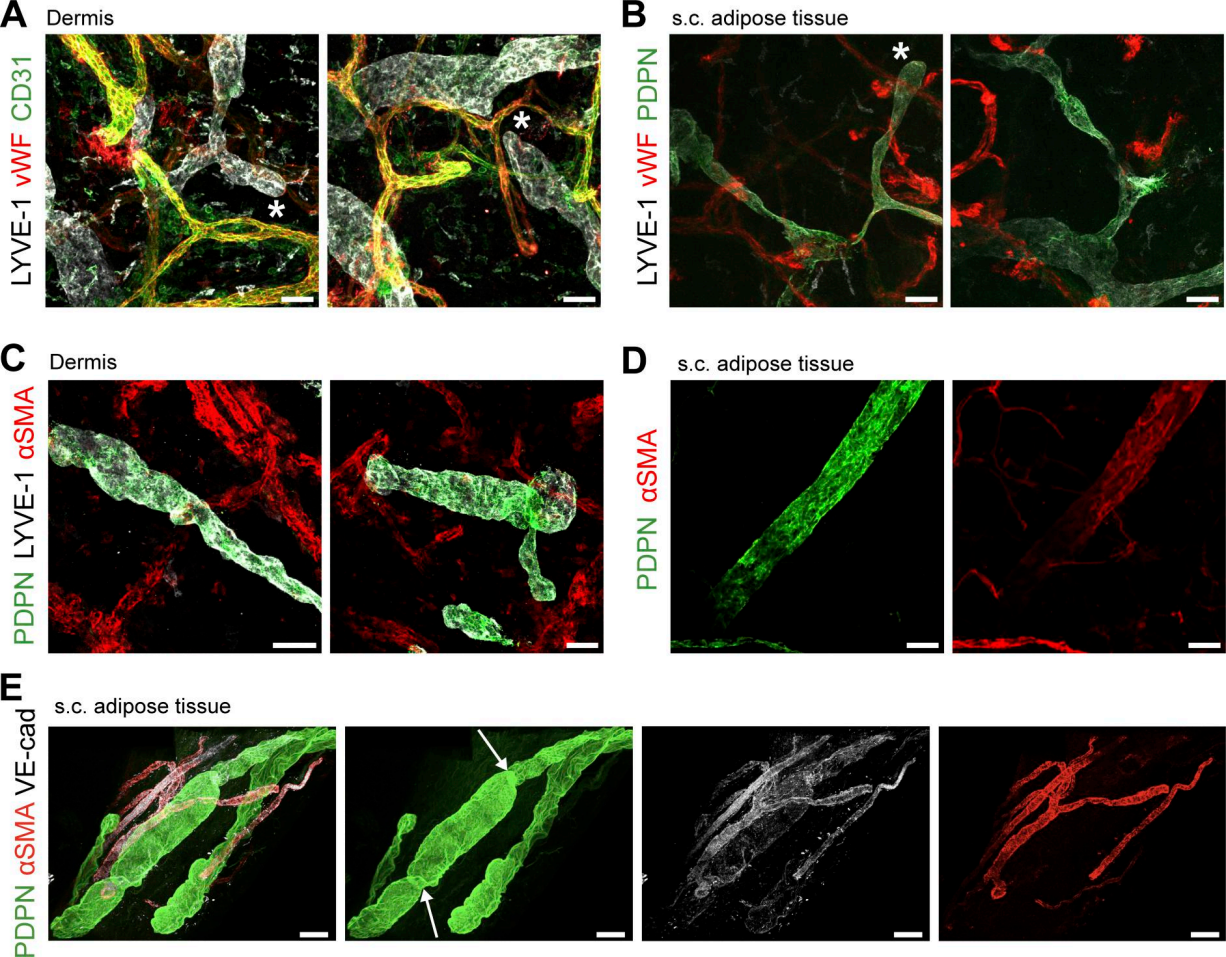
**3D visualization of LVs in human dermis and s.c. adipose tissue by confocal microscopy. (A and B)** Confocal images of (A) human dermis (upper 200 µm) and (B) s.c. adipose tissue showing LVs and blind-ended lymphatic capillaries (depicted by *), as well as BVs (vWF^+^). PDPN: Podoplanin. **(C and D)** Confocal images of (C) human dermis (upper 200 µm) depicting lymphatic capillaries (PDPN^+^LYVE-1^+^aSMA^−^) and (D) human adipose tissue showing a lymphatic collector (PDPN^+^aSMA^+^). A representative image of >10 experiments (A) is shown. One lymphatic collector (PDPN^+^aSMA^+^) was observed in 10 whole mounts of adipose tissue (B and D) and none in >10 whole mounts of dermis (C). Scale bars (A–D): 50 µm. **(E)** Representative confocal image of human s.c. adipose tissue immunostained for PDPN, aSMA, and VE-cadherin (*n* = 4 donors). The picture represents a Tilescan of multiple stitched images. The white arrows highlight valve-like structures. Scale bar: 100 µm.

**Figure S1.**
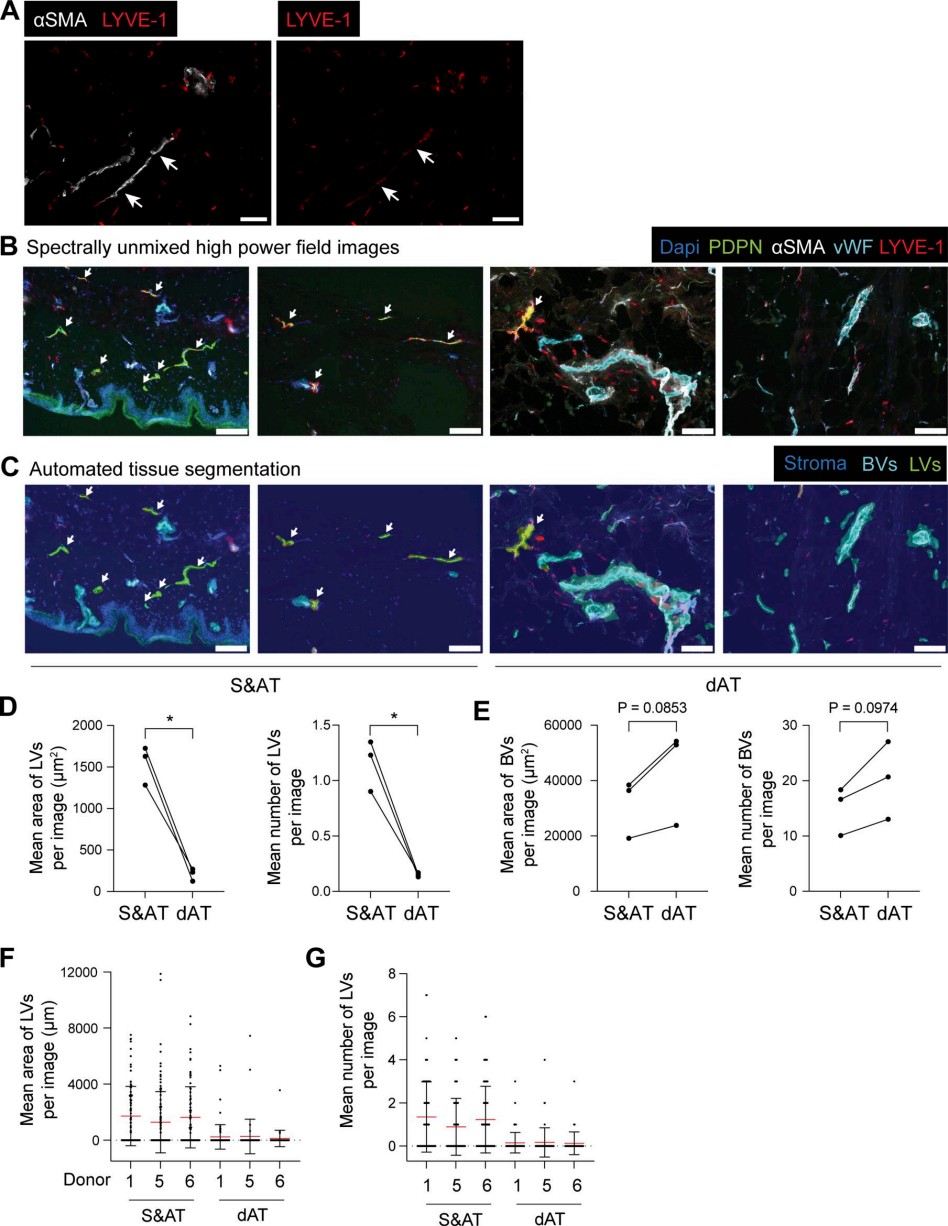
**LVs are more abundant in the skin and adjacent s.c. adipose tissue than in the deeper adipose tissue. (A)** aSMA^+^ LYVE-1^+^ lymphatic collectors in adipose tissue (depicted by arrows). Only two lymphatic collectors were observed in this set of images. Scale bar: 100 µm. **(B)** Representative examples of spectrally unmixed high-power field images for sections of S&AT, as well as dAT. Scale bars: 100 µm. **(C)** Corresponding automated tissue segmentation results, highlighting the detection of LVs, BVs, and the surrounding tissue (stroma). LVs in B and C are depicted by an arrow. Scale bars: 100 µm. **(D and E)** Quantification of the (D) mean area (µm^2^) or number of LVs and (E) BVs for *n* = 3 donors. One dot represents the mean of the quantification of all images for one donor per tissue type. Data from the same donor are connected by a line and analyzed by paired Student’s *t* test. *P < 0.05. **(F and G)** Quantification of the (F) mean area (µm^2^) or (G) number of LVs. Mean and standard deviation are shown separately for each donor (one dot represents one image). For each tissue, sections of three different depths were evaluated. Images were acquired by multispectral microscopy from a representative set of S&AT and dAT regions (12–47 images per tissue and patients, total of 451 images). S&AT, human skin and attached s.c. adipose tissue; dAT, deeper s.c. adipose tissue.

**Figure 2.**
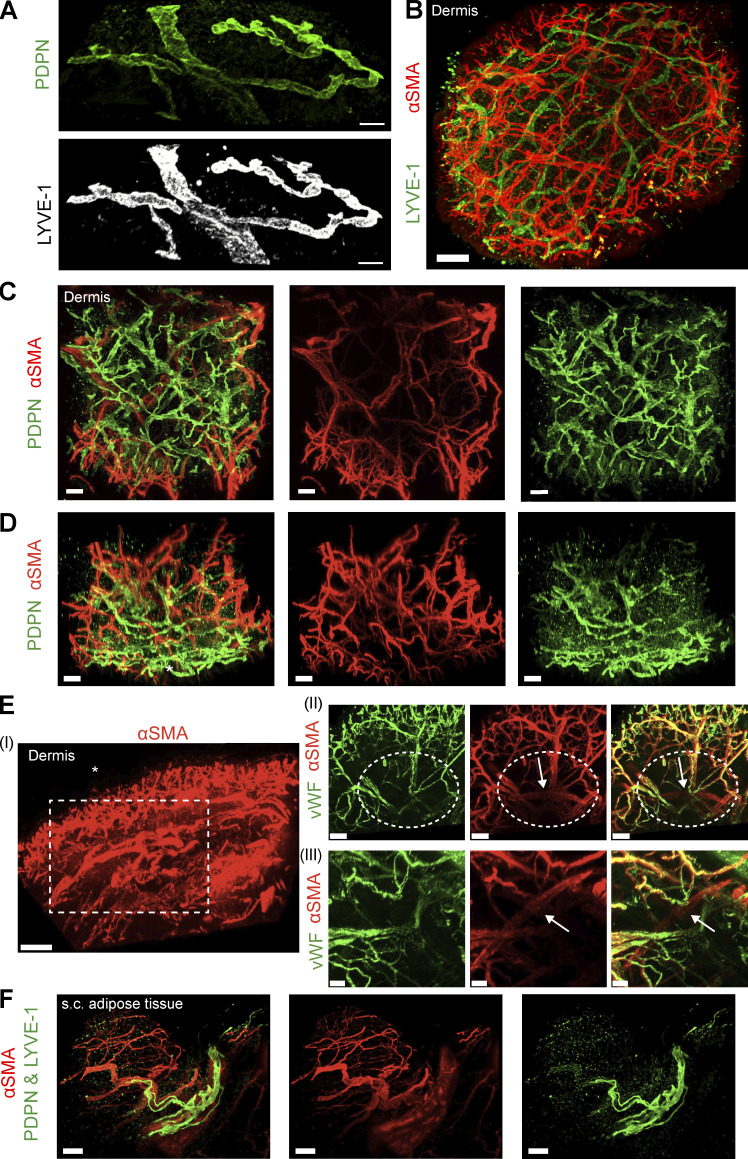
**Light-sheet microscopy images of aSMA-covered vasculature in human skin and s.c. adipose tissue. (A–** **E** **)** Light-sheet microscopy images of cleared and immunostained biopsies of human dermis. Images show (A) PDPN^+^ LYVE-1^+^ LVs, but no PDPN^+^LYVE-1^−^ LVs, and (B) LYVE-1^+^aSMA^−^ LVs and aSMA^+^ BVs. (C–E) Representative images of cleared and immunostained biopsies of human dermis acquired by light-sheet microscopy (*n* = 6–10 donors per condition). C and D show PDPN^+^aSMA^−^ LVs and aSMA^+^ BVs, and D shows a side view of the skin with the asterisk * indicating the location of the epidermis. Note that D is the same image as the one shown in C (rotated by 90°). 3D views of B–D are provided in Videos 1 and 2. Images in B–D were acquired through the epidermis with a stack height (i.e., penetration depth) of 2.2 and 1.8 mm, respectively. **(E)** Skin whole mount stained for aSMA and vWF. (I) Overview of the entire skin piece imaged with the superficial and deeper vascular plexus visible, imaged from the side to reveal the two vascular plexi. The asterisk (*) indicates the location of the epidermis. Scale bar: 400 µm. (II) Magnification of the squared angle marked with a dotted line in I, imaged from the bottom of the sample, i.e., from a different orientation. Scale bar: 200 µm. (III) Magnification of the circled area marked with a dotted line in II, zooming in on an aSMA^+^vWF^−^ vessel likely representing a dermal lymphatic collecting vessel. Scale bar: 100 µm. Note that this was the only aSMA^+^vWF^−^ vessel detected in >6 immunostained punches from 6 donors. A 3D view of E is provided in Video 3. **(F)** Light-sheet microscopy images of cleared and immunostained biopsies of human s.c. adipose tissue. PDPN^+^ or LYVE^+^ LVs (both antibodies detected in the same color) running along larger aSMA^+^ BVs. Representative images of >2/3 (experiments/donors) (A), >3/6 (experiments/donors) (B), 5 (C and D), and >10 (F) experiments are shown. Scale bars: (A) 100 µm, (B) 500 µm, (C and D) 150 µm, (F) 100 µm. Tissue source in light-sheet images shown: breast (A, B, and E), thigh (C and D), and abdomen (F).

To better study the morphology of human afferent LVs, we established a protocol to perform 3D confocal imaging in punch biopsies of the upper 200 µm of human dermis and in a s.c. adipose tissue. Staining for the lymphatic markers Podoplanin (PDPN) and LV endothelial hyaluronan receptor 1 (LYVE-1), in combination with the BV marker von Willebrand factor (vWF), we observed many initial, blind-ended lymphatic capillaries in close proximity to BVs (vWF^+^) in the human dermis and in s.c. adipose tissue (Fig. 1, A and B). In the skin, virtually all LVs were negative for alpha-smooth muscle actin (aSMA), which is expressed in vascular smooth muscle cells surrounding BVs and lymphatic collectors (Fig. 1 C), indicating the low abundance of collectors in the skin. Rarely, lymphatic collectors (aSMA^+^) were found in adipose tissue (Fig. 1, D and E; and Fig. S1 A). Generally, LVs were ∼10-fold less frequent in s.c. adipose tissue compared with skin, as determined by quantification of 2D sections (Fig. S1, B–G). Also, BVs were 10–20 times more abundant in tissue sections as compared to LVs (Fig. S1, D–E). To further investigate the 3D organization and network of human afferent LVs, we performed light-sheet microscopy on immunostained, optically cleared full-thickness skin punch biopsies. This approach enabled imaging through the epidermis to a depth of up to 2.2 mm into the dermis (Fig. 2, A–E; and Videos 1, 2, and 3). Surprisingly, and in contrast to findings in murine skin (Arasa et al., 2021; Pflicke and Sixt, 2009), we consistently detected only PDPN^+^LYVE-1^+^ LVs but not PDPN^+^LYVE-1^−^ LVs in human dermis (Fig. 1 C and Fig. 2 A). Moreover, in agreement with our confocal imaging (Fig. 1 C) and analysis of 2D sections (Fig. S1, B–G), LVs in human full-thickness skin, identified by either LYVE-1 or PDPN staining, were typically aSMA-negative, indicating low abundance of lymphatic collectors (Fig. 2, B–D; and Videos 1 and 2). In line with this assessment, we observed a near-complete overlap of aSMA staining with the BV marker vWF; only in one case out of >6 different biopsies stained did we detect an aSMA^+^ vWF^−^ vessel in the deep dermis, which likely represented a lymphatic collector (Fig. 2 E and Video 3). Similarly, light-sheet imaging of cleared and immunostained s.c. adipose tissue biopsies revealed that LVs were generally lacking LMC coverage (Fig. 2 F). Taken together, these findings indicated that lymphatic collectors are rare in both tissues.

**Video 1.** **LYVE-1** ^**+**^ **LVs in green and aSMA** ^**+**^ **vessels in red in a punch biopsy of human breast skin imaged by light-sheet microscopy.** Of note, also some LYVE-1^+^ macrophages can be observed. The video corresponds to Fig. 2 B. Scale bar: 500 µm.

**Video 2.** **PDPN** ^**+**^ **LVs in green and aSMA** ^**+**^ **vessels in red in a punch biopsy of human thigh skin imaged by light-sheet microscopy.** The video corresponds to Fig. 2, C and D. Scale bar: 400 µm.

**Video 3.** **vWF** ^**+**^ **BVs in green and aSMA** ^**+**^ **vessels in red in a punch biopsy of human breast skin imaged by light-sheet microscopy.** The video corresponds to Fig. 2 E. Scale bar: 300 µm.

### Seven different LEC subsets are present in human skin and s.c. adipose tissue

**Figure 3.**
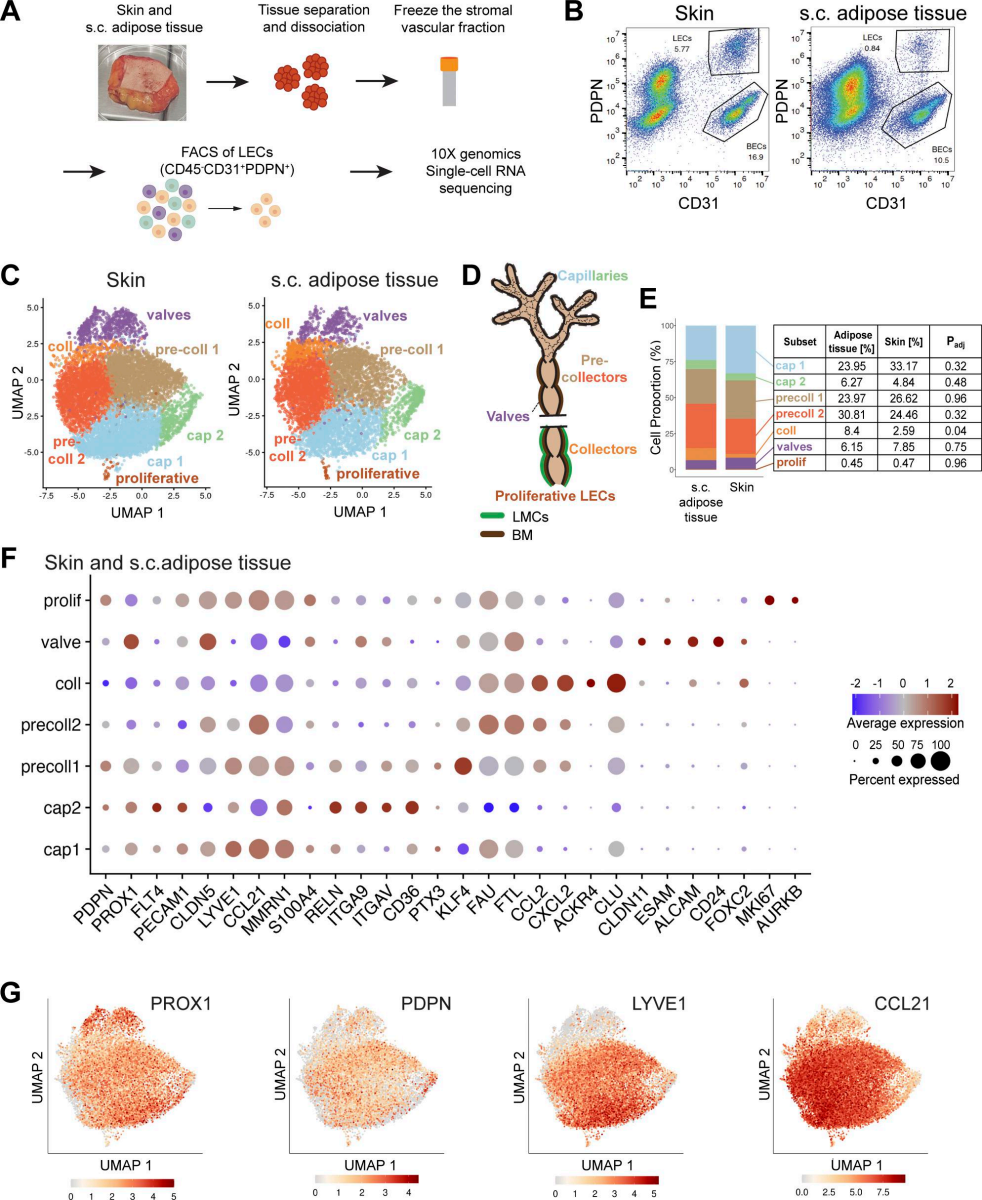
**scRNA-seq of LECs derived from human skin and adipose tissue reveals the presence of seven subsets of LECs. (A)** Schematic overview of LEC isolation process from human skin or adipose tissue for scRNA-seq (seven donors). **(B)** Representative flow cytometry plot of the isolated frozen stromal vascular fraction from skin and adipose tissue showing a population of LECs (CD45^−^CD31^+^PDPN^+^). The full gating scheme can be found in Fig. S2, A and B. **(C)** Seven LEC clusters from human skin or adipose tissue visualized in a UMAP plot. **(D)** Schematic of a LV depicting the different subsets of LECs as detected in the scRNA-seq data. BM: basement membrane. **(E)** Representation of the proportions and percentages of each LEC subset present in the skin and adipose tissue. The adjusted P value (P_adj_) of the differential abundance of LEC clusters between both tissue types was computed using the Bioconductor/R edge R package. Collector LECs were significantly more abundant in adipose tissue compared with skin (P_adj_ < 0.05). **(F)** Bubble plot showing the expression of selected pan-EC, pan-LEC, and LEC subset marker genes in the seven subsets. Expression data from the skin and adipose tissue combined are shown. The color and size of each dot represent the expression level and cell fraction of the indicated genes, respectively. **(G)** UMAP plots showing the expression of specific LEC marker genes, i.e., *PROX1*, *PDPN*, *LYVE1*, *CCL21*. Abbreviations: cap: capillary; precoll: pre-collector; coll: collector; prolif: proliferative.

**Figure S2.**
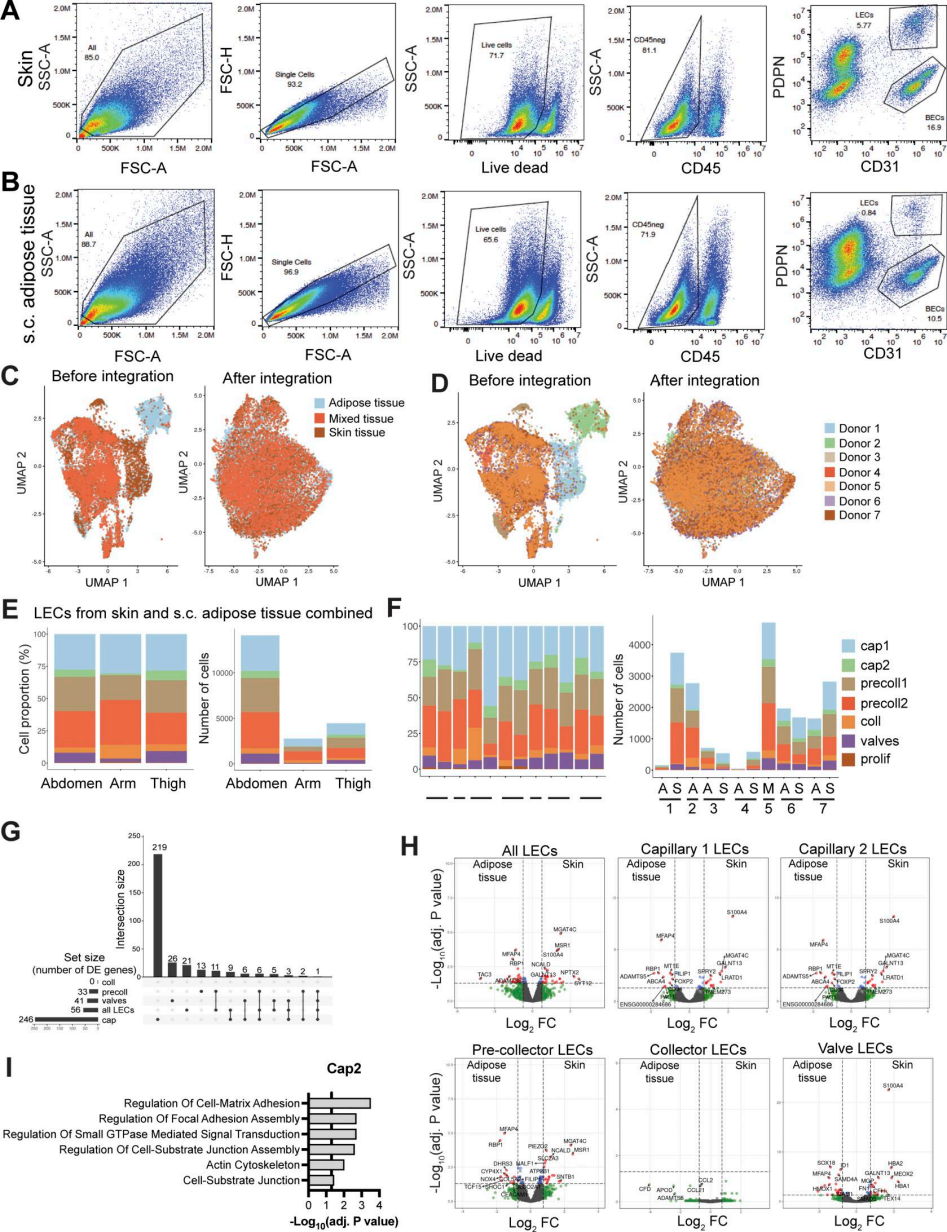
**Similar gene expression profile in LECs from adipose tissue and skin. (A and B)** Representative flow cytometry plots of the isolated stromal vascular fraction from (A) the skin and (B) the adipose tissue showing a population of LECs (CD45^−^CD31^+^PDPN^+^). The figure shows the complete gating scheme, with the final gating plot also presented in a simplified form in Fig. 3 B. **(C and D)** UMAP plots before and after removing batch effect by integration visualized according to (C) the tissue or (D) the donor. Donor 5 is a mixture of skin- and adipose tissue–derived LECs that cannot be differentiated between tissue types (mixed tissue). **(E)** Proportion (%) and cell numbers for each LEC subset according to the tissue origin. **(F)** Proportion (%) and cell numbers for each subset according to the donor and the tissue type (adipose tissue [A], skin [S], mixed [M]). **(G)** UpSet plot showing overlaps in DEG (FDR < 0.05, log_2_FC >0.75) between all major clusters (skin vs. adipose tissue) ranked by the overlap with the largest number of genes. The right chart shows the intersection size of the conditions highlighted in the middle grid. A single, unconnected point corresponds to genes unique to only that condition. The total number of DEGs in each cluster is shown in the left chart (set size). **(H)** Volcano plot of DEGs between adipose tissue and skin of different LEC clusters or all LECs. The following LEC clusters were considered for matched tissue donors (i.e., donors 1, 3, 6, 7): capillary 1, capillary 2, pre-collector (merged precoll1 and precoll2 subsets), collector, and valve LECs. The horizontal line shows the log_2_ fold change (FC) threshold set at 0.75. The vertical lines show the significance threshold for adjusted P values set at an FDR of 0.05. **(I)** GO term analysis of genes enriched in skin cap2 LEC subset compared with the s.c. adipose tissue cap2 LEC subset. Selected terms for enriched GO biological and cellular processes are shown along with the −log_10_ of the adjusted P value (list of marker genes for the enrichment analysis in Data S4). The vertical line represents the adjusted P value set at an FDR of 0.05. FDR, false discovery rate.

**Figure S3.**
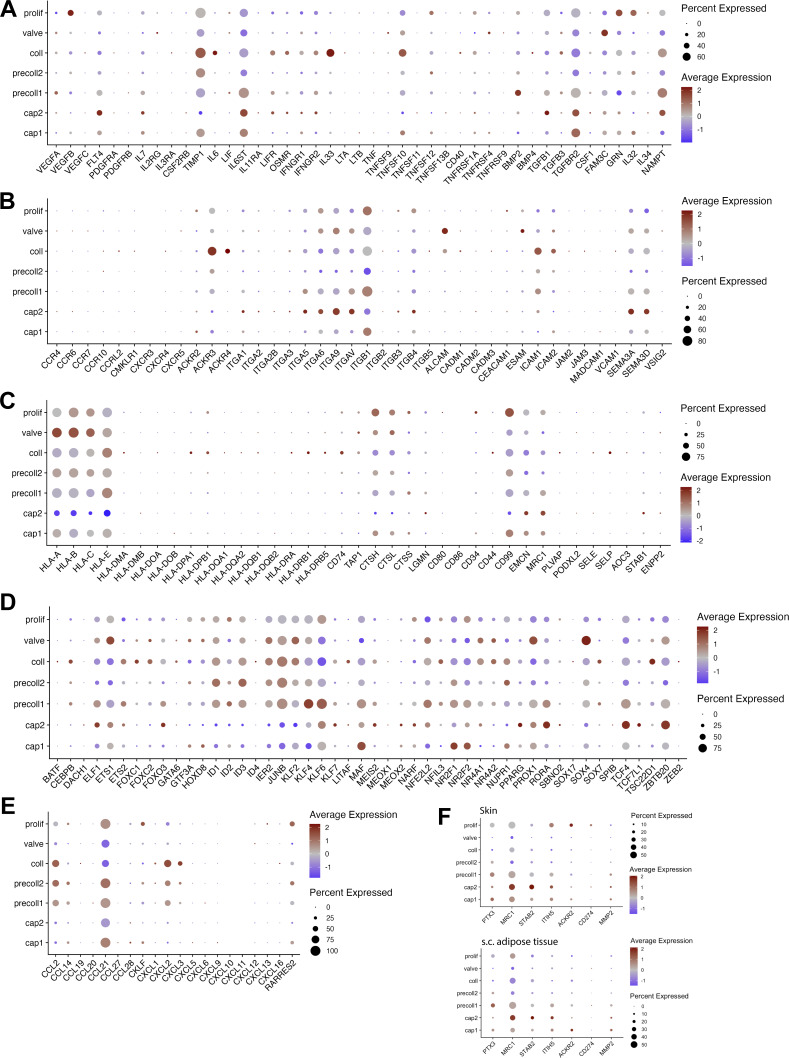
**Expression of various molecules in all LEC subsets identified in the scRNA-seq dataset. (A–C)** Bubble plots of the seven dermal LEC subsets showing the expression of selected genes encoding (A) cytokines, growth factors, and their receptors, (B) molecules involved in cell adhesion and migration, and (C) molecules involved in antigen presentation and the immune response. **(D–F)** Transcription factors (D), chemokines (E), and genes (F) recently found to be expressed by immune-interacting *Ptx3*^*+*^ LECs present in murine skin (Petkova et al., 2023). Only data from the skin are shown in A–E, whereas a comparison of skin and s.c. adipose tissue data is shown in F. The color and size of each dot represent the expression level and cell fraction of the indicated genes, respectively.

To further investigate the heterogeneity of LECs present in human afferent LVs, we performed scRNA-seq of LECs isolated from human skin or from s.c. adipose tissue. To this end, fresh tissue samples were obtained from elective dermolipectomy surgeries performed on the abdomen, arm, or thigh of healthy, albeit obese patients. Following manual separation of the skin from the underlying s.c. adipose tissue and subsequent tissue digestion, we performed FACS-based cell sorting on single-cell suspensions to isolate live CD45^−^CD31^+^PDPN^+^ LECs from each tissue (Fig. 3, A and B; Fig. S2, A and B; and Table S1). LECs were isolated from seven donors (Table S1), and subjected to scRNA-seq using 10x Genomics, which yielded a total of 21,374 sequenced cells (9,417 from skin, 7,252 from s.c. adipose tissue, and 4,705 unassigned to either origin; see Table S1 for details). Unsupervised clustering revealed seven subsets of LECs in both skin and adipose tissue–derived samples, which we assigned as capillary, pre-collector, collector, valve, and proliferative subsets, based on previously reported lymphatic marker gene expression (Arasa et al., 2021; Gonzalez-Loyola et al., 2021; Hernandez Vasquez et al., 2021) and relative positioning on the UMAP (Fig. 3, C–E; and Table S2 for the top 20 cluster markers). All LECs derived from skin and s.c. adipose tissue expressed the pan-endothelial genes *PECAM1* (*CD31*) and *CLDN5* and the lymphatic markers *PDPN, PROX1*, and *FLT4* (*VEGFR-3*) (Fig. 3, F and G; and Fig. S3, A–E). Most subsets appeared relatively close in the UMAP plot, i.e., shared overlapping gene signatures; however, the most distinctive subsets were the valve and the proliferative LECs. The latter represented only 0.4% of all LECs and was identified based on the expression of the proliferation markers *MKI67* and *AURKB* (Fig. 3, C–F). Notably, a similar subset was also reported in recent scRNA-seq studies of LECs derived from mouse mesentery (Gonzalez-Loyola et al., 2021) or mouse skin (Petkova et al., 2023). Valve LECs expressed the valve marker and tight junction protein *CLDN11* (Ortsater et al., 2021), connexin-37 (*GJA4*) and connexin-43 (*GJA1*) (Kanady et al., 2011; Sabine et al., 2012), and the transcription factor forkhead box protein C2 (*FOXC2*) (Norrmen et al., 2009; Petrova et al., 2004). The identity of the collector subset was confirmed by the expression of *ACKR4* (Fig. 3 F), a chemokine scavenging receptor which we and others recently found to be upregulated by flow in LMC-covered murine collecting vessels (Friess et al., 2022; Redder et al., 2023, *Preprint*). For the two capillary (cap1, cap2) subsets, two pre-collector (precoll1, precoll2) subsets, and the collector subset, very few subset-specific genes were found, suggesting that the subset transition occurs in a continuous manner. Nevertheless, a few distinguishing features of these subsets could be identified. Lymphatic capillaries expressed high levels of the capillary marker *LYVE1* and the chemokine *CCL21*, which were progressively diminished in pre-collectors, collectors, and valves. Unlike a previous report (Wick et al., 2008), pre-collector LECs in our scRNA-seq dataset neither exhibited reduced *PDPN* expression nor expressed *CCL27* (Fig. 3, F and G; and Fig. S3 E). However, we did observe a specific chemokine signature in the pre-collector and collector subsets. The chemokines *CCL2* and *CXCL2*, chemoattractants for T cells or monocytes and neutrophils, respectively, were both found at the pre-collector level and highly expressed in the collectors (Fig. 3 F and Fig. S3 E). In mice, a *PTX3*^*+*^ immune-interacting dermal LEC subset localizing at the tips of lymphatic capillaries was recently identified (Petkova et al., 2023). When analyzing the expression of *PTX3* and other immune-interacting LEC signature genes (e.g., *MRC1*, *ACKR2*, and *ITIH5*), we observed a broad expression of these signature genes across the precoll1 and capillary subsets (Fig. S3 F). While *PTX3* was more prominent in precoll1 subset, other genes that define the signature of immune-interacting LECs, such as *MRC1*, *STAB2*, and *ITIH5*, were specific for cap2 both in the skin and in the adipose tissue (Fig. S3 F). Moreover, *PTX3* was only expressed at low levels in human dermal LECs (Fig. S3 F).

In general, the proportions of the seven subsets remained relatively constant across different tissue origins and among various donors (Fig. S2, E and F). The precoll1 and precoll2 subsets combined constituted more than half of the LECs in skin (51%) and in s.c. adipose tissue (55%), followed by the combined cap1 and cap2 subsets (30% and 38%, respectively). There were strikingly more collector LECs in the s.c. adipose tissue compared with the skin (8.4% and 2.6%, P_adj_ = 0.04, Fig. 3 E). However, despite this increase in collector LECs, the proportion of valve LECs remained comparable between the two tissues (7.9% in s.c. adipose tissue and 6.2% in skin, Fig. 3 E). Consequently, the valve/coll LEC ratio was more than fourfold higher in the skin (3.02) compared with s.c. adipose tissue (3.02 vs. 0.73, Fig. 3 E). In agreement with our analysis of 2D sections (Fig. S1, B–G), confocal and light-sheet microscopy only rarely detected lymphatic collectors (aSMA^+^) in s.c. adipose tissue or in the skin (Figs. 1 and 2).

### Capillary LECs from skin and adipose tissues exhibit subtle transcriptional differences

To examine the gene composition and variations in LECs based on their tissue origin, we conducted a pseudobulk differential expression analysis comparing skin and adipose tissue-derived LECs from matched tissue donors. We observed that LECs from skin and adipose tissue were very homogeneous with only 56 differentially expressed genes (DEGs) (Fig. S2 G). No significant differences in the expression of major LEC marker genes between skin and adipose tissue were detected (e.g., *CCL21*, *LYVE1*, *ACKR4*). When looking at individual LEC clusters, no DEGs were identified in collector LECs and only a few in the combined pre-collector and the valve clusters, highlighting the transcriptomics similarity of these clusters in the skin and adipose tissue (Fig. S2, G and H). The combined capillary clusters had the most DEGs, indicating that cap1 and cap2 LECs were the subsets that varied the most between the skin and adipose tissue (Fig. S2 G). Interestingly, *S100A4*, a protein recently found to be produced by LECs and to promote sprouting during tumor-associated lymphangiogenesis (Li et al., 2023), emerged as one of the most strongly upregulated genes in the skin in both the cap1 and cap2 subsets and also in valve LECs (Fig. S2 H). Moreover, two glycosyltransferases, namely, *GALNT13* and *MGAT4C* were highly upregulated in dermal cap1 and cap2 LEC subsets, suggesting potential differences in glycosylation between capillary LECs from skin and s.c. adipose tissue (Fig. S2 H). Conversely, all LEC subsets from s.c. adipose tissue, except for collector LECs, overexpressed *MFAP4*, a glycoprotein of the ECM recently implicated in angiogenesis (Schlosser et al., 2025). Overall, a high transcriptional similarity between skin and s.c. adipose tissue LECs was found, with subtle tissue-specific signatures emerging primarily in the two capillary subsets.

### Plasticity of LEC morphology and junctions in dermal initial capillaries

**Figure 4.**
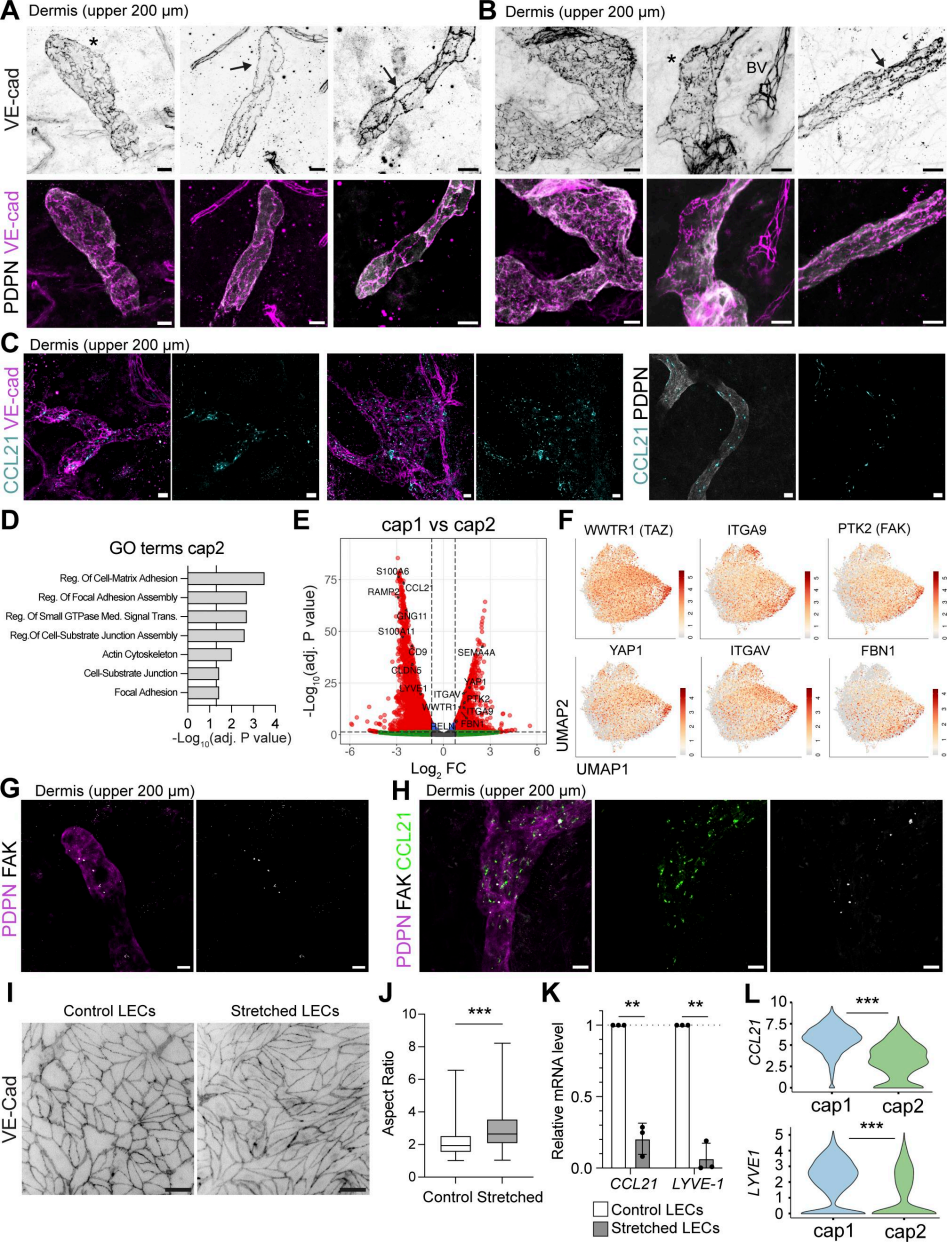
**Mixed junctional state in capillary LV. (A and B)** Whole mounts prepared from the upper 200 µm of the human dermis showing PDPN^+^ LVs and the distribution of the junctional protein VE-cadherin (VE-cad). Representative images from *n* = 8 donors are shown. **(A)** Blind-ended lymphatic capillaries with oak leaf–shaped LECs (depicted by an asterisk) or more elongated LECs (depicted by an arrow) were observed in human dermis. **(B)** Images of LVs with oak leaf–shaped LECs joined by button junctions (depicted by an asterisk) or LVs with more elongated, zipper-like LECs (depicted by an arrow), as well as BVs joined with zipper junctions. Scale bars: 20 µm. **(C)** Whole mounts of human dermis (upper 200 µm) showing the presence of intracellular CCL21 in afferent LVs stained with anti-VE-cadherin or anti-PDPN antibodies. Representative images from *n* = 3 donors are shown. Scale bars: 20 µm. **(D)** GO term analysis of genes enriched in the cap2 LEC subset compared with all other LEC subsets. Selected terms for enriched GO biological and cellular processes are shown along with the −log_10_ of the adjusted P value (list of marker genes for the enrichment analysis in Data S4). The vertical line represents the adjusted P value set at an FDR of 0.05. **(E)** Volcano plot of DEGs between cap1 and cap2 LEC subsets. The horizontal line shows the log_2_ fold change (FC) threshold set at 0.75. Vertical lines show the significance threshold for adjusted P values set at an FDR of 0.05. **(F)** Individual UMAP plots showing the expression of specific cap2 LEC genes, i.e., *WWTR1*, *ITGA9*, *PTK2*, *YAP1*, *ITGAV*, *FBN1*. **(G and H)** Whole mounts prepared from the upper 200 µm of the human dermis showing PDPN^+^ LVs expressing (G) the focal adhesion kinase (FAK), and (H) FAK and the chemokine CCL21. Representative images from *n* = 3 donors are shown (G and H). Scale bars: 20 µm. For the FAK images, a thin stack at the level of the LV was made to visualize FAK expression specifically in LECs. **(I)** Human dermal LECs were subjected to mechanical stretch (10% strain every 30 s for 18 h) with a bioreactor. Representative immunofluorescence images of control (static) human dermal LECs and human dermal LECs subjected to stretching are shown. VE-cadherin was used to visualize the cell boundary and subsequent elongation. Scale bar: 100 µm. **(J)** Aspect ratio of control LECs or stretched LECs was quantified and is represented as a box plot. Statistics were computed with the nonparametric Mann–Whitney test. Pooled data were derived from *n* = 3 independent replicates with 695 cells analyzed in total. ***P < 0.001. **(K)** RNA of control or stretched human dermal LECs was extracted, and RT-qPCR was performed. The fold change expression levels of *CCL21* and *LYVE1* between stretched and control samples are depicted. A fold change below one shows reduced gene expression under stretched conditions. Two independent experiments from *n* = 3 human dermal LEC donors, with four pooled technical replicates for each. Statistics were computed with paired Student’s *t* test. **P <0.01. **(L)** Violin plots showing differential expression of *CCL21* and *LYVE1* in the cap1 and cap2 clusters. Statistics: the P adjusted value is shown. ***P <0.001. FDR, false discovery rate.

From whole-mount immunofluorescence studies performed primarily in murine skin or trachea, we know that LECs of initial lymphatics of uninflamed mouse tissues are joined by dynamically remodeled, discontinuous button-like junctions, which act as primary valves for fluid and cell entry into lymphatics (Baluk et al., 2007; Pflicke and Sixt, 2009; Schoofs et al., 2025). However, to the best of our knowledge, these junctions have not been imaged in human tissue. Using the endothelial junction protein VE-cadherin to visualize the morphology of LECs in whole mounts of human dermis, we could clearly observe button-like junctions and the oak leaf shape of LECs in initial lymphatics imaged in the upper 200 µm of human dermis (Fig. 4, A and B, depicted by an asterisk). Conversely, in other cases, we also found blind-ended capillaries and LVs composed of elongated LECs that were connected by zipper-like junctions (Fig. 4, A and B, depicted by an arrow). Thus, these findings uncover mixed junctional states at the level of initial lymphatics in human skin.

### Specialized ECM-binding capillary subset

Capillary LECs from both the skin and s.c. adipose tissue clustered into two populations in our scRNA-seq data; i.e., the main capillary subset, cap1, accounting for 33% and 24% of all LECs, and a minor subset of cap2 LECs, corresponding to 4.8% and 6.3% of all LECs in skin and s.c. adipose tissue, respectively (Fig. 3, C–F). The cap1 subset was characterized by high expression of the capillary markers *CCL21* and *LYVE1* (Fig. 3, F and G). In accordance with our whole-mount analyses detecting LYVE-1 in all LVs in human dermis (Fig. 1, A and C; and Fig. 2 A), our scRNA-seq data indicated *LYVE1* expression in all LEC subsets except for the collector and valve LEC subsets (Fig. 3, F and G). Similarly, besides its expression in cap1, CCL21 was mainly expressed in pre-collectors (precoll1, precoll2) (Fig. 3, F and G). The presence of intracellular CCL21 in LVs and blind-ended capillaries was confirmed by whole mounts of human dermis (Fig. 4 C). Intriguingly, the cap2 subset displayed strikingly reduced levels of *LYVE1* and *CCL21* at the mRNA level, yet high expression of the pan-LEC transcripts *PDPN, PROX1*, and *FLT4* (Fig. 3, F and G). Analysis of the DEGs from the cap2 LEC subset compared with all other LECs revealed enrichment in pathways associated with terms such as cell–matrix adhesion, focal adhesion assembly, cell–substrate junction, and focal adhesion (Fig. 4 D). Interestingly, the same or similar pathways were upregulated when comparing the cap2 subset from skin to that from s.c. adipose tissue, suggesting that these pathways are particularly prominent in the skin (Fig. S2 I). The cap2 subset was generally enriched for the capillary marker *RELN* (Lutter et al., 2012) and several integrins (*ITGAV*, *ITGA9*), suggesting that cap2 cells might be actively involved in the binding to the ECM (Fig. 4, E and F). Cap2 LECs also expressed fibrillin (*FBN1*), which is the main component of anchoring filaments (Fig. 4, E and F) (Gerli et al., 2000; Weber et al., 2002). It is currently unknown whether all capillary LECs bind to anchoring filaments or only a certain subset. The fact that several molecules involved in mechanosensing or mechanosignal transduction, such as focal adhesion kinase (*PTK2* or *FAK*) and downstream effectors including *YAP1* and *TAZ* (*WWTR1*), were upregulated in cap2 LECs suggests a mechano-activated state of this subset (Fig. 4, E and F). We validated the expression of FAK in LVs by 3D confocal imaging performed in the upper 200 µm of human dermis (Fig. 4, G and H). To investigate the mechano-activated state of the cap2 cluster, we subjected primary human dermal LECs to *in vitro* stretching using a dedicated bioreactor (Bachmann et al., 2016). As previously reported in BECs (Bernardi et al., 2018), stretched human dermal LECs responded to the mechanical stimulus by elongating (Fig. 4, I and J). When analyzing the mRNA expression of *CCL21* and *LYVE1* in stretched human dermal LECs, we observed that these LEC markers were significantly downregulated in comparison with the unstretched control (Fig. 4 K), in line with their significant downregulation in the cap2 compared with the cap1 subset in our scRNA-seq data (Fig. 4 L). Overall, these observations suggest the presence of a small mechanoactive capillary subpopulation that is specialized in ECM binding in human tissues.

### High proportion of valves present in LVs of the upper dermis

**Figure 5.**
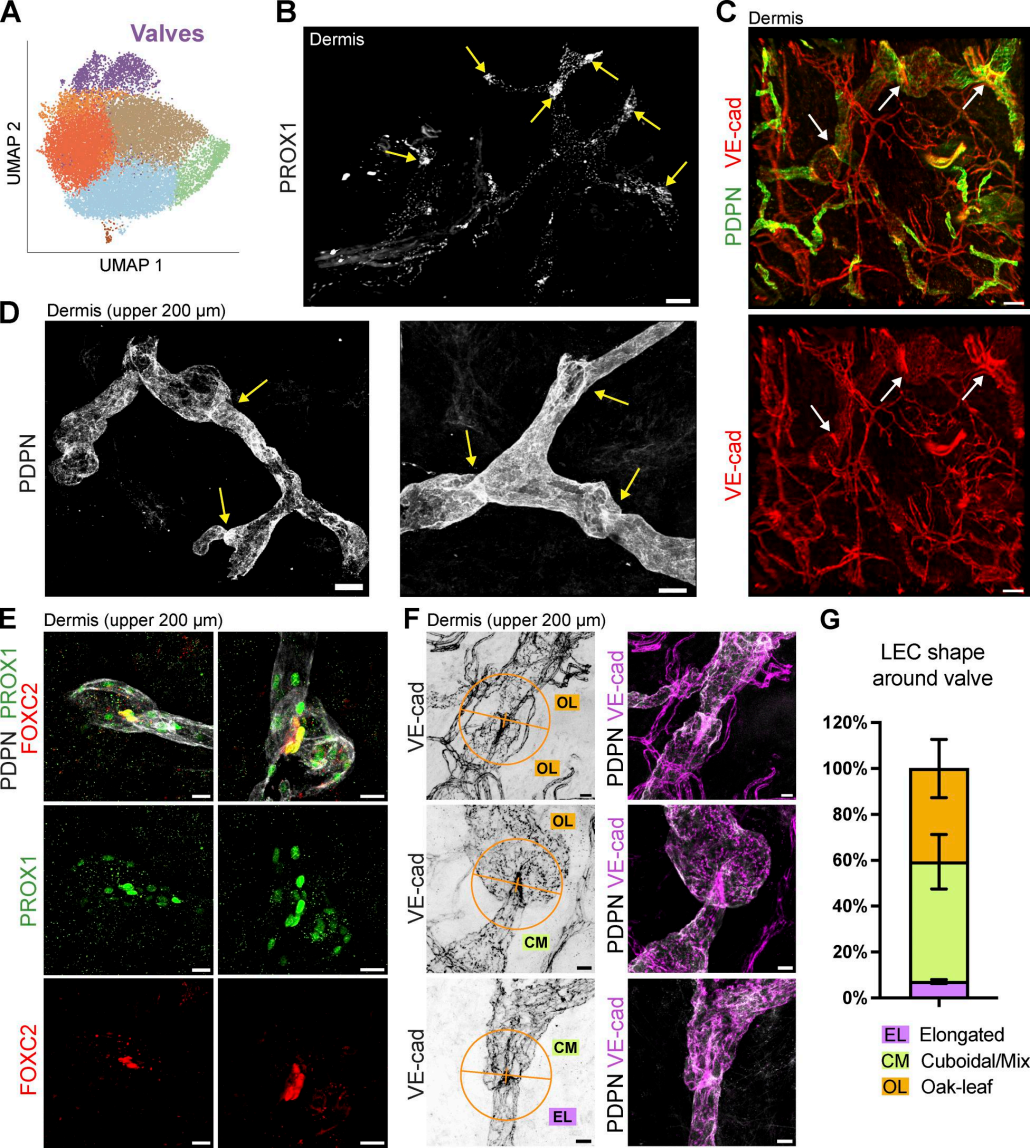
**Lymphatic valves are present in human dermis. (A)** UMAP plot of skin and adipose tissue combined showing the proportion (in %) of valves in each tissue type (skin and adipose tissue). **(B)** Cleared and immunostained biopsy of whole-thickness human dermis imaged by light-sheet microscopy, showing the presence of condensed PROX1^+^ areas representing lymphatic valves (depicted by arrows). Scale bar: 100 µm. **(C)** Cleared and immunostained biopsy of human dermis imaged by light-sheet microscopy showing the presence of condensed VE-cadherin regions in valve-like regions (depicted by arrows) of PDPN^+^ LVs. Scale bars: 100 µm. Representative images from *n* > 7/8 (experiments/donors) (B) and *n* > 2/4 (experiments/donors) (C) are shown. Source of skin in B and C: abdomen. **(D)** Whole-mount images of the upper 200 µm of the human dermis showing valve-like structures (depicted by arrows) in PDPN-expressing LVs. Scale bar: 50 µm. **(E)** Representative images from *n* = 3 donors showing the colocalization of PROX1^+^FOXC2^+^ LECs in lymphatic valves in the upper 200 µm of human dermis visualized by immunofluorescence of whole mounts. Scale bars: 20 µm. **(F and G)** Quantification of LEC morphology around lymphatic valve regions. Human skin punches were costained for VE-cadherin and PDPN, and LVs, valves, and LEC shape were visualized in cleared samples by confocal microscopy or light-sheet imaging. LEC shape was assessed in a 60-µm radius (half a circle) spanning the upstream and downstream areas of lymphatic valves. LECs were categorized into three morphological types: oak leaf (OL), cuboidal or mixed (CM), or elongated (EL). **(F)** Representative images illustrating the different morphological categories. Scale bars: 20 µm. **(G)** Quantification of LEC shape around valves. A total of 28 valves (50 valve regions) from six donors were analyzed by three independent analyses (D and F). Results are shown as percentages of all valve areas analyzed.

Despite the scarcity of collecting vessels (Fig. 1, C−E and Fig. 3 C), the valve LEC subset made up for a relatively high proportion of all LECs in our scRNA-seq analysis, namely, 6.2% and 7.8% in the skin and s.c. adipose tissue, respectively (Fig. 3 E and Fig. 5 A). In the mouse, valves within lymphatic collectors reportedly express specific markers such as claudin-11 (CLDN11), integrin-α9 (ITGA9), and FOXC2 (Bazigou et al., 2009; Ortsater et al., 2021; Petrova et al., 2004). In addition, valves can be visualized as condensed PROX1^+^ areas, due to elevated *PROX1* expression in valve LECs and valves harboring a high LEC density (Hagerling et al., 2017; Norrmen et al., 2009). Indeed, performing light-sheet microscopy of cleared dermal punches, we observed numerous areas of condensed PROX1 signal, revealing the presence of lymphatic valves (Fig. 5 B). The presence of valves in human dermis was also confirmed by staining for VE-cadherin, which was previously found to condensate in valve areas (Sabine et al., 2018) (Fig. 5 C). Surprisingly, we frequently observed valve-like structures within the first 200 µm of dermis and even near blind-ended capillaries (Fig. 5 D). These valves in the upper dermis co-expressed PROX1 and the transcription factor FOXC2, further confirming their valve identity (Fig. 5 E). We further observed that valves in the upper human dermis were frequently surrounded by oak leaf–shaped LECs with clearly visible button-like junctions, as determined by staining for VE-cadherin (Fig. 5 F). Quantification of the shape of LECs surrounding lymphatic valves in the human dermis confirmed the presence of mostly oak leaf–shaped or mixed (cuboidal)-shaped but rarely elongated LECs (Fig. 5 G). Thus, lymphatic valves in the upper human dermis are found in LVs with a pre-collector phenotype that are characterized by the absence of LMC coverage (Fig. 1 C and Fig. 2, B–D) and with LECs frequently displaying an oak leaf– or mixed-shaped morphology and button-like cell–cell junctions.

### Valve LECs fall into two subsets with unique gene expression profiles

**Figure 6.**
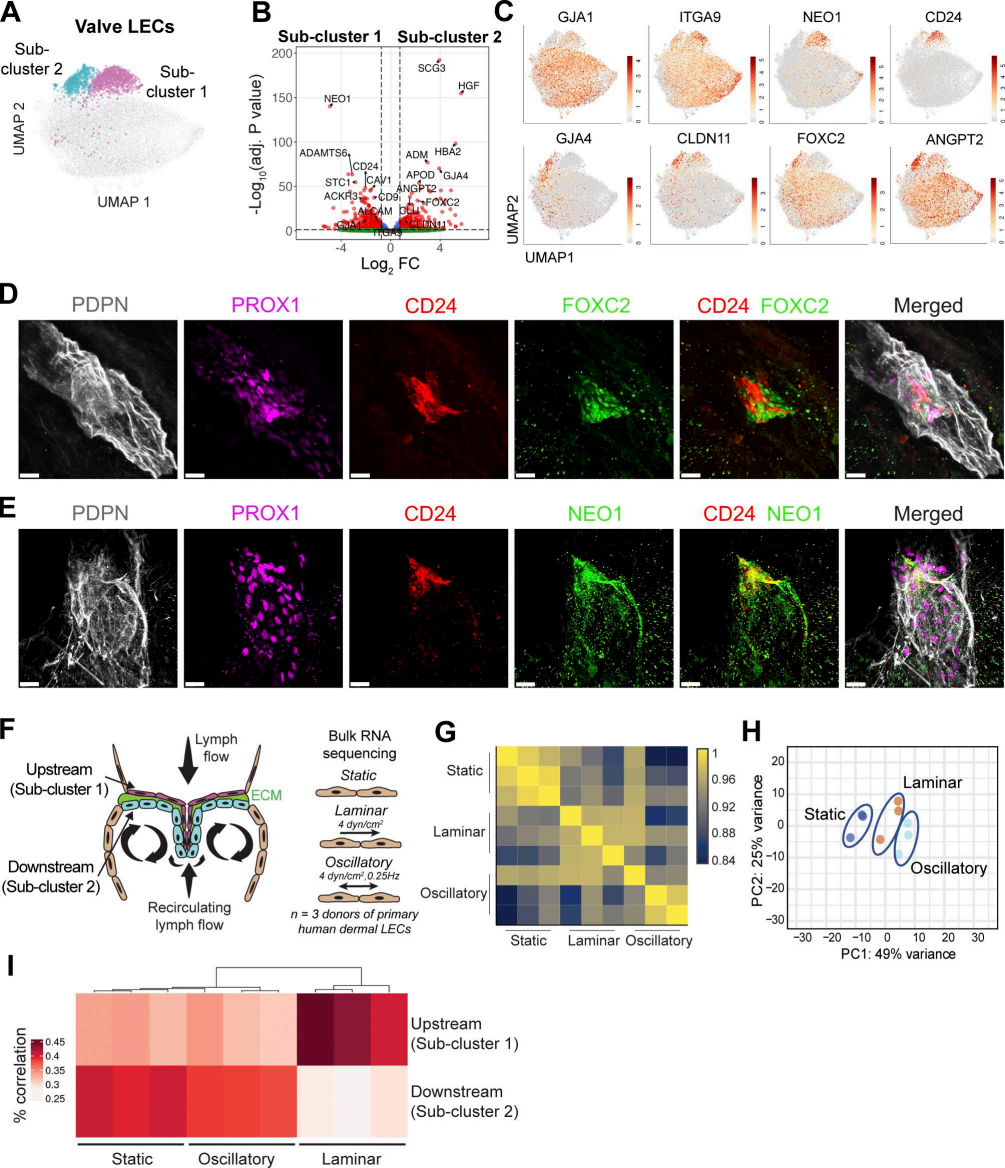
**Valve population is composed of two subclusters with different gene expression. (A)** Subclustering analysis of the valve cluster revealed two valve subclusters, corresponding to the LECs on the upstream sides of the valve leaflets, and LECs on the downstream sides of the valve leaflets. **(B)** Volcano plot of DEGs between the upstream valve LEC and downstream valve LEC clusters. The horizontal line shows the log_2_ fold change (FC) threshold set at 0.75. Vertical lines show the significance threshold for adjusted P values set at an FDR of 0.05. **(C)** Individual UMAP plots showing the expression of DEGs in upstream valve LECs (*GJA1*, *ITGA9*, *NEO1*, *CD24*) and in downstream valve LECs (*GJA4*, *CLDN11*, *FOXC2*, *ANGPT2*). **(D and E)** Confocal analysis of valves present in the upper 200 µm of human dermis confirmed (D) the differential expression of FOXC2 and CD24 by LECs on different sides of the valve leaflet, and (E) the colocalization of CD24 and NEO1 by LECs on the same valve leaflet side. Representative images from *n* = 2–3 independent experiments (donors) are shown in D and E. Scale bars: 20 µm. **(F)** Schematic illustration of the structure of the valves and the positioning of the LECs within the valves, based on Saygili Demir et al. (2023). **(G)** Schematic illustration of bulk RNA-seq of human dermal LECs subjected to laminar shear stress (4 dyn/cm^2^), oscillatory-like shear stress (laminar shear stress at 4 dyn/cm^2^ that reverses direction by 180° every 4 s), or static conditions for 48 h. The top 50 DEGs in the laminar and oscillatory conditions are shown in Data S2 and S3. **(G)** Pearson correlation plot of the bulk RNA-seq described in E. **(H)** PCA plot of the samples described in G analyzed at three different conditions (static, oscillatory, and laminar). **(I)** Similarity of LEC clusters upstream and downstream of valves with the bulk RNA-seq of human dermal LECs subjected to static conditions; laminar or oscillatory flow was determined by the correlation of the top 100 DEGs of valve LEC clusters (Data S1) with the bulk RNA-seq dataset. FDR, false discovery rate.

**Figure S4.**
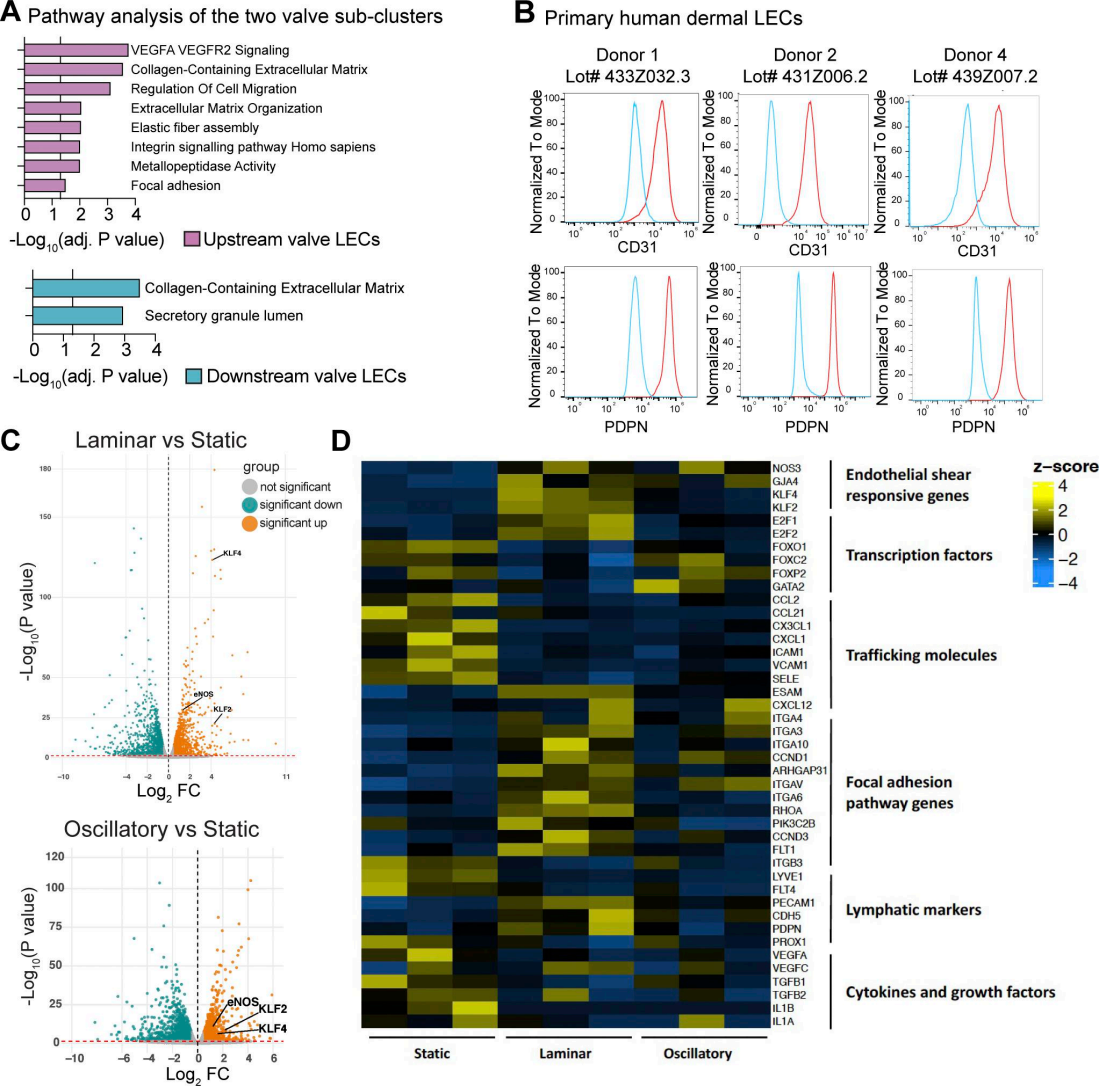
**Bulk RNA-seq of human dermal LECs subjected to laminar or oscillatory shear stress. (A)** Selected pathways from Enrichr analysis (GO terms) of DEGs upregulated in the LECs on the upstream sides of valve leaflets or upregulated in LECs on the downstream sides of valve leaflets (list of DEGs used for the enrichment analysis in Data S5). The vertical line represents the adjusted P value set at an FDR of 0.05. **(B)** Flow cytometry analysis of CD31 and PDPN expression of the three human dermal LEC donors used for bulk RNA-seq. Cells were gated on single cells, followed by viable cells, and the expression histograms shown in this panel. **(C)** Volcano plots of significant DEGs when comparing laminar or oscillatory shear stress to static control. **(D)** Heatmap of expression levels of selected genes in the bulk RNA-seq study. FDR, false discovery rate.

Since the valve subset appeared to contain two distinct populations (Fig. 5 A), we performed subclustering of the valve population, which revealed two lymphatic valve subclusters (subclusters 1 and 2, Fig. 6 A) that displayed very distinct gene expression profiles (Fig. 6 B and Data S1). Interestingly, the two valve LEC subclusters differentially expressed connexin-43 (*GJA1/CX43*) and connexin-37 (*GJA4/CX37*), which were previously found to be expressed in LECs on the upstream and on the downstream sides of murine valve leaflets (Kanady et al., 2011; Sabine et al., 2012), suggesting that the *GJA1*-expressing subcluster 1 represented LECs on the upstream and the *GJA4*-expressing subcluster 2 represented LECs on the downstream sides of the valve leaflets (Fig. 6, B and C). In line with this assessment, *FOXC2*, which is upregulated by oscillatory flow in LECs of the downstream murine valve leaflet (Sabine et al., 2015), was preferentially expressed in subcluster 2. LECs on the upstream sides of the valve leaflets further expressed DEGs such as *ITGA9*, *neogenin 1* (*NEO1*), and *CD24*, whereas LECs on the downstream sides of the valve leaflets expressed *CLDN11* and *ANGPT2* (Fig. 6, B and C). A pathway enrichment analysis of the DEGs revealed pathways associated with terms such as integrin signaling, ECM organization, and cell migration for the upstream valve LECs, and collagen-containing ECM for the downstream valve LECs (Fig. S4 A). Performing 3D confocal imaging in the upper 200 µm of the human dermis, we confirmed the preferential and differential expression of CD24 and FOXC2 in LECs of distinct valve leaflets (Fig. 6 D). Conversely, NEO1 (Fig. 6, B and C) and CD24 colocalized on the same valve leaflet (Fig. 6 E). Together, these findings confirmed NEO1 and CD24 as novel markers of LECs forming the upstream valve leaflet.

### Shear stress partially explains the distinct gene expression patterns of the two valve subsets

LECs in valves are exposed to rather high, fluctuating shear stress conditions that arise during the valve opening and closing (Moore and Bertram, 2018; Urner et al., 2018). Consequently, they need to adhere tightly to each other and to the ECM core sandwiched between two layers of LECs that form the valve leaflet (Fig. 6 F). It has been postulated that LECs on the two sides of the valve leaflets are exposed to different levels and types of shear force: LECs on the upstream side are thought to be directly exposed to the laminar flow created by the incoming lymph, whereas LECs on the downstream side presumably are exposed to lower and more disturbed, oscillatory flow (Fig. 6 F) (Angeli and Lim, 2023; Pujari et al., 2020; Sabine et al., 2015; Sabine et al., 2016). To assess how shear stress influences the gene expression signature of the two valve LEC subsets, we performed a bulk RNA-seq on *in vitro* cultured human dermal LECs isolated from three different donors (Fig. S4 B). Specifically, the cells were subjected to unidirectional laminar shear stress (4 dyn/cm^2^), oscillatory-like shear stress (consisting of laminar shear stress at 4 dyn/cm^2^ that reverses direction by 180° every 4 s), or static conditions (Fig. 6, G–I; Fig. S4, C and D; and Data S2 and S3). When comparing the top 100 DEGs between the two valve clusters in the scRNA-seq (Data S1) to the bulk RNA-seq data, we observed a better correlation of the gene expression signature of LECs in the upstream subcluster 1 with the gene expression of LECs subjected *in vitro* to laminar as compared to oscillatory-like shear stress or static (no flow) conditions (Fig. 6 I). In contrast, the gene expression signature of LECs present in the downstream subcluster 2 correlated better with the gene expression signature of LECs subjected to oscillatory-like shear stress and static conditions than to that of LECs subjected to laminar shear stress, although this correlation was less pronounced (Fig. 6 I). Similarly, analysis of the top 10 DEGs between the two valve clusters (Fig. 6 B) revealed that genes enriched in the upstream valve LEC subcluster were highly upregulated upon *in vitro* exposure of dermal LECs to laminar flow, whereas those associated with the downstream subcluster were predominantly downregulated (Table S3). Again, this correlation was markedly weaker under oscillatory-like flow conditions *in vitro* (Table S3), suggesting that our *in vitro* setup did not fully recapitulate the turbulent flow characteristic of the downstream valve leaflet compartment *in vivo*. Overall, these findings suggested that shear stress induced by laminar flow partially accounted for the distinct gene expression profiles of the two valve subsets found in human LECs in the dermis and adipose tissue.

### CD24 is a human and murine valve marker and contributes to valve development in the mesentery

**Figure 7.**
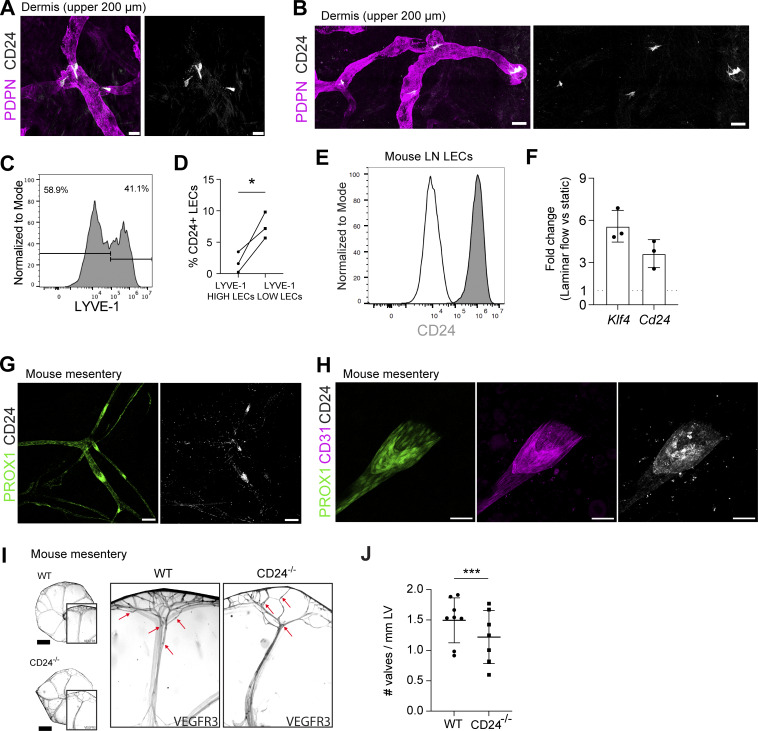
**Characterization of CD24 as a novel lymphatic valve marker in human and mouse. (A and B)** Whole mounts prepared from the upper 200 µm of human dermis, showing the expression of CD24 at valve-like regions in PDPN^+^ LVs. The picture shown in B represents a Tilescan of multiple stitched images. Scale bars: left, 50 µm; right, 100 µm. Representative images from >10 independent experiments (donors). **(C and D)** Flow cytometry–based analysis of CD24 expression in LYVE-1^low^ and LYVE-1^high^ LECs (CD45^−^CD31^+^PDPN^+^) in the stromal vascular fraction of human dermis. **(C)** Representative histogram plot showing the gating of LYVE-1^low^ and high LECs. **(D)** Quantification of the percentage (%) of CD24-expressing LECs in LYVE-1^low^ and LYVE-1^high^ LECs from three independent experiments (*n* = 3 donors). Each dot represents one independent experiment. Statistics were computed with paired Student’s *t* test. *P < 0.05. **(E)** CD24 expression in mouse LN LECs. Representative flow cytometry plots of three experiments. **(F)** LN LECs were subjected for 48 h to laminar flow (Lam, 4 dyn/cm^2^) or static conditions. RT-qPCR was performed on the extracted mRNA. The fold change is shown, and values above 1 represent genes upregulated under shear stress. Each dot represents one independent experiment performed with LN LECs from different isolations (*n* = 3). **(G and H)** Whole mounts of *Prox1-*eGFP mouse mesentery showing expression of (G) CD24 in lymphatic valves visualized with Prox1 (high in valves) and (H) in combination with the cell adhesion molecule CD31. Representative images from five independent experiments (G and H). Scale bars: (G) 150 µm, (H) 50 µm. **(I and J)** Quantification of mesenteric valves in *Cd24*^*−/−*^ or WT pups. In each experiment, mesenteries were collected from 4- to 5-day-old littermate pups (WT *Cd24*^+/+^ and *Cd24*^−/−^) obtained from *Cd24*^+/−^ × *Cd24*^+/−^ crosses. Valves were quantified using whole-mount histology. **(I)** Representative images of WT and *Cd24*^*−/−*^ mesenteries showing VEGFR3 staining. Some valves are indicated by a red arrow. Note that for the identification of valves, the combination of several markers was used (see Materials and methods). Scale bar: 2 mm. **(J)** Quantification of the valve numbers in mesenteric vessels in WT (^+/+^) and *Cd24*^−/−^ mesenteries. The absolute number of valves per mm LV is shown. In each experiment, all WT (^+/+^) and *Cd24*^−/−^ mesenteries from littermates of a *Cd24*^+/−^ × *Cd24*^+/−^ crossing were analyzed. In total, pups from six litters containing at least one pup from each genotype were analyzed. Each dot represents the value from one pup. Statistics: linear mixed-effects model with litter as a random effect to account for within- and between-litter variation. ***P < 0.001.

Among the genes differentially expressed between the two valve subclusters, the adhesion molecule *CD24* (Fig. 6, B–E) stood out as it was among the most upregulated genes within the entire LEC valve subset in our scRNA-seq analysis (Data S1). CD24 has thus far not been investigated in the context of LEC biology; however, it was found among valve-specific transcripts in recent mouse and human scRNA-seq studies (Gonzalez-Loyola et al., 2021; Kumar et al., 2023; Petkova et al., 2023; Takeda et al., 2019). In addition to its expression in LECs of the upstream valve leaflet (Fig. 6, D and E), CD24 proved to be a particularly robust valve marker in human skin, remaining clearly detectable in confocal immunostainings of the upper dermis even at lower magnifications (Fig. 7, A and B). In line with the valve-specific expression of CD24 observed by confocal microscopy, flow cytometry performed on human dermal single-cell suspensions identified a significantly higher percentage of CD24-expressing LECs when gating on LYVE-1^low^ LECs, which comprise valves, as compared to when gating on LYVE-1^high^ LECs (Fig. 7, C and D).

**Figure S5.**
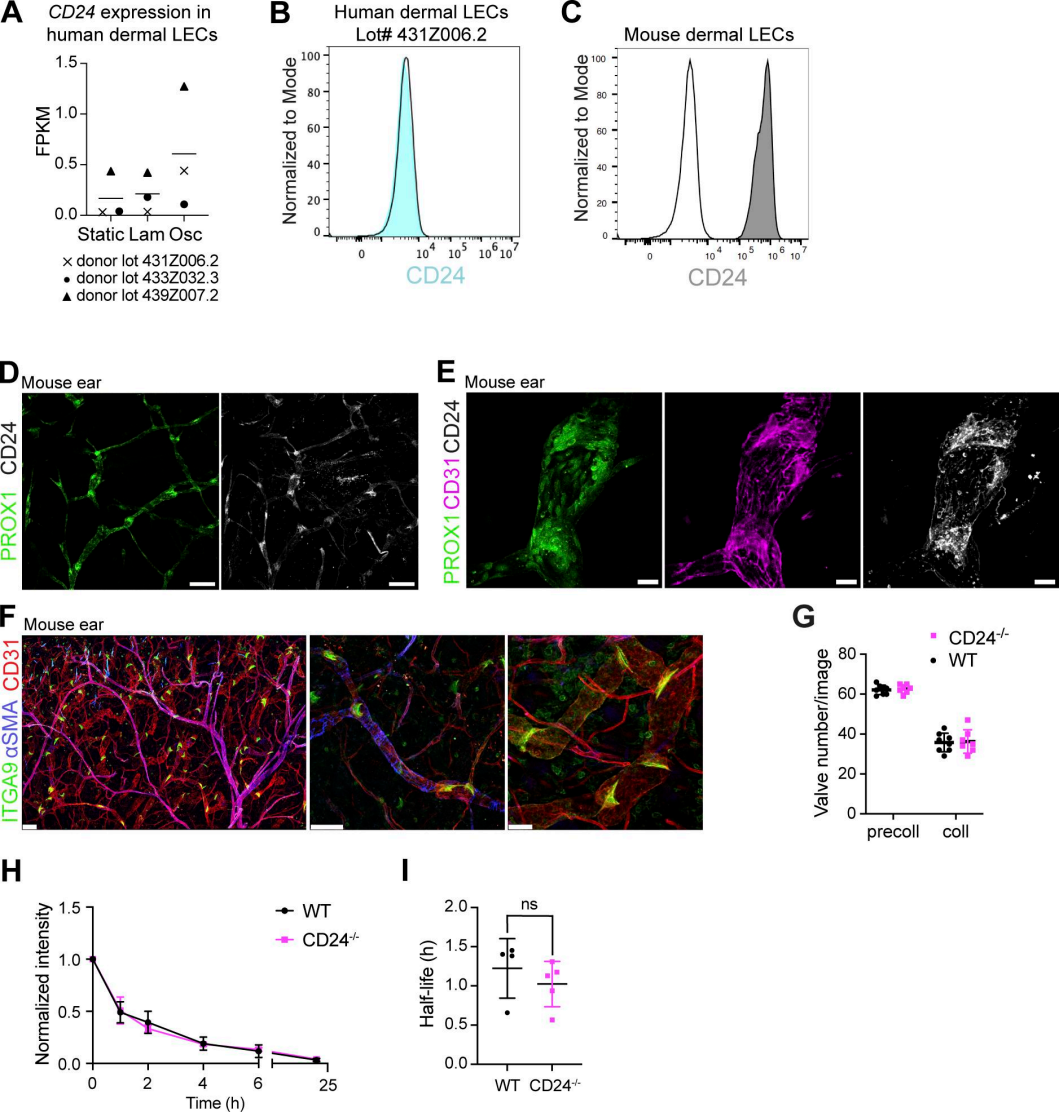
**CD24 expression in murine and human dermal LECs *in vitro* and *in vivo*. (A)** RNA expression in FPKM in three donors of human dermal LECs subjected to laminar (lam) shear stress, oscillatory (osc) shear stress, or static conditions (data from the bulk RNA-seq). **(B)** CD24 is not expressed at the protein level in *in vitro*–cultured human dermal LECs as assessed by flow cytometry. Representative flow cytometry plot of donor 431Z006.2. Donors 433Z032.3 and 439Z007.2 neither expressed CD24. **(C)** CD24 expression in mouse dermal imLECs. Representative flow cytometry plot of three experiments. **(D and E)** Whole mounts of *Prox1-*eGFP mouse ear showing expression of (D) CD24 in lymphatic valves visualized with Prox1 (high in valves) and (E) in combination with the cell adhesion molecule CD31. Representative images from five independent experiments. Scale bars: (D) 100 µm, (E) 25 µm. **(F and G)** Quantification of ITGA9^+^ valves was performed in 6-image Tilescans acquired in an ear skin area containing predominantly collectors (coll: CD31^+^aSMA^+^) or pre-collectors (precoll: CD31^+^aSMA^−^). **(F)** Representative image of a Tilescan (left) and of ITAGA9^+^ valves in CD31^+^aSMA^−^ pre-collecting (middle) and CD31^+^aSMA^+^ collecting vessels (right). Scale bars from left to right: 150, 100, 50 µm. **(G)** Quantifications of valves in the pre-collector or collector area of ear skin whole mounts from adult WT (*Cd24*^+/+^) and *Cd24*^−/−^ mice (*n* = 8 WT and *n* = 7 *Cd24*^−/−^). **(H and I)** Lymphatic drainage assay: adult WT and *Cd24*^−/−^ mice were injected with a near-infrared dye conjugate intradermally in the ear skin. Clearance of the tracer was monitored over 24 h by IVIS imaging. **(H and I)** (H) Average dye clearance plots and (I) calculated half-lives in WT and *Cd24*^*−*/−^ mice (data from one experiment with four to five mice per group are shown). ns, not significant; FPKM, fragments per kilobase of transcript per million mapped reads.

As *in vitro*–cultured human dermal LECs showed virtually no CD24 expression at either the RNA or the protein level (Fig. S5, A and B), CD24 was not detected among the flow-induced genes in our bulk RNA-seq analysis (Table S3 and Fig. S5 A). In contrast to its absence in cultured human dermal LECs, CD24 was detectable by flow cytometry in *in vitro*–cultured murine LN LECs and in dermal immortalized LECs (imLECs) (Vigl et al., 2011) (Fig. 7 E and Fig. S5 C). To investigate the flow responsiveness of *Cd24* in the murine system, we exposed murine LN LECs to laminar shear stress. Under these conditions, *Cd24* was upregulated together with the known flow-responsive gene *Klf4* (Clark et al., 2011), confirming the flow responsiveness of *Cd24* (Fig. 7 F). We next investigated the expression of CD24 in murine lymphatic valves by whole-mount immunofluorescence in mouse mesentery, which primarily contains collecting LVs (Sabine et al., 2018), as well as in mouse ear skin, which contains capillaries but also pre-collectors and aSMA-covered collectors (Arasa et al., 2021; Friess et al., 2022; Hernandez Vasquez et al., 2021). In both tissues, CD24 was expressed in lymphatic valves, and its expression colocalized with the known upregulation of the lymphatic-specific transcription factor PROX1 in the valve region (Fig. 7, G and H; and Fig. S5, D and E). In line with CD24 expression in mouse erythrocytes, nerves, and certain immune cells (e.g., B cells, transiently on T cells) (Altevogt et al., 2021; Ayre and Christian, 2016), CD24 was also detected in further cell types and structures in the tissue.

To investigate the potential involvement of CD24 in valve development, we quantified the number of mesenteric lymphatic valves in 4- to 5-day-old *Cd24*^*−*/−^ pups and wild-type (WT) littermates. *Cd24*^−/−^ pups exhibited a significant reduction in mesenteric valve numbers compared with WT controls (Fig. 7, I and J), indicating its functional role in lymphatic valve development in the mesentery. On the other hand, when investigating valve numbers and lymphatic drainage in adult mouse ear skin, we observed no differences in either parameter between WT and *Cd24*^*−*/−^ mice (Fig. S5, F–I). Taken together, our results identify CD24 as a robust, flow-induced valve marker and reveal its contribution to lymphatic valve development in the murine mesentery.

## Discussion

The composition and organization of the lymphatic vasculature in human tissues is only beginning to be fully understood. In this study, combining 3D imaging and scRNA-seq, we identified several differences between the human and murine afferent lymphatic vasculature of the skin. We also identify CD24 as a new lymphatic valve marker and show that it is functionally important for the formation of lymphatic valves in the mesentery.

In murine skin, LYVE-1^−^ collecting vessels make up a sizable fraction of the lymphatic vasculature (Arasa et al., 2021; Friess et al., 2022; Hernandez Vasquez et al., 2021; Pflicke and Sixt, 2009). By comparison, in human skin whole mounts, LYVE-1 was expressed in virtually all LVs, revealing its broader expression pattern in human as compared to murine skin. In line with this observation, <10% of all dermal LECs in our scRNA-seq dataset were LYVE-1^−^, corresponding to either collector LECs (2.6%) or valve LECs (6.2%). Unlike murine skin, and consistent with the low percentage of collector LECs, we found little evidence of LMC-covered LVs during 3D imaging of full-thickness human skin analyzed at depths of up to 2.2 mm. The scarcity of LMC-covered lymphatic collectors in human dermis is surprising, given that human skin is much thicker than murine skin, potentially leading to greater blood vascular leakage and lymphatic drainage demands.

Our observation that LYVE-1^+^ pre-collector LECs are the most prevalent LEC subset in human skin suggests that pre-collectors may play a critical role in maintaining dermal tissue fluid homeostasis and supporting lymphatic drainage. In line with this assumption, we identified many valve LECs, which localized to pre-collecting vessels as shown by 3D imaging. Intriguingly, EM studies performed >40 years ago by Daroczy already reported the presence of valves in dermal LVs, which at the time were identified as capillaries based on morphological features like the absence of neighboring LMCs (Daroczy, 1983; Daroczy, 1984). Since we gathered only limited evidence for the presence of aSMA^+^ LVs, corresponding to collectors, in full-thickness human skin, we conclude that most valve-containing vessels are pre-collectors. Our findings confirm and extend a recent light-sheet microscopy–based study that also reported the presence of PROX1^+^ valves in human dermal PDPN^+^ LVs (Hagerling et al., 2017). In the latter and in our study, valves were found to be present already in the upper dermis and even in proximity to blind ends of capillaries. The fact that we frequently observed valves in vessel segments composed of oak leaf–shaped LECs, which are believed to be specialized in fluid and leukocyte uptake, is particularly intriguing and might suggest that valves exert functions that extend beyond fluid transport. For example, intravital microscopy studies performed in murine skin have revealed that leukocytes in lymphatic capillaries actively patrol and migrate, since fluid flow in this compartment is not strong enough to sustain their passive transport (Collado-Diaz et al., 2022; Russo et al., 2016; Teijeira et al., 2014). It is therefore possible that valves in dermal pre-collectors, which are also present in murine skin (Fig. S5, F and G), might additionally function to guide the intraluminal, active migration of leukocytes from one lymphangion to the next, in the direction of LMC-covered lymphatic collectors, from where rapid, flow-mediated transport to dLNs occurs.

Murine LECs in lymphatic capillaries have traditionally been described as oak leaf–shaped and connected by characteristic button-like cell–cell junctions, thereby generating entry routes for interstitial fluid, macromolecules, and immune cells (Baluk et al., 2007). Intriguingly, our 3D imaging experiments revealed the substantial junctional and cell morphological diversity of initial human dermal capillaries, which comprised button junctions, zipper junctions or intermediate junctions, and more elongated LECs alongside oak leaf–shaped LECs in blind-ended capillaries. In mice, junctional plasticity of initial lymphatics has particularly been observed during development, inflammatory conditions, or infections, when button-like junctions reportedly transform into zippers (Baluk et al., 2007; Churchill et al., 2022; Yao et al., 2012; Zhang et al., 2018). One possible explanation of the mixed junctional and cell shape phenotype observed in the human skin might therefore be the diverse history of the human skin analyzed in our study in terms of age and disease status (e.g., previous infections, other underlying pathologies, or poorer skin quality in case of previous massive weight loss) of the donors, which contrasts with the skin collected from mice bred in standardized and highly hygienic conditions. Mixed junctional phenotypes in lymphatic capillaries have recently also been reported in steady-state murine skin (Schoofs et al., 2025) and nasal mucosa (Hong et al., 2023), highlighting the plasticity and complexity of lymphatic junctions in both mice and humans.

Besides morphological differences, we also observed several gene expression difference between murine and human LEC subsets: for example, human dermal collector LECs displayed an activated phenotype, as judged from the strong expression of chemokines (*CXCL2*, *CXCL3 CCL2*), *ACKR3*, *ICAM-1*, or *IL33* (Fig. S3, A, B, and E), which is not observed in published murine dermal collector LEC datasets (Arasa et al., 2021; Petkova et al., 2023). Conversely, the immune-interacting *Ptx3*^*+*^ subset (Petkova et al., 2023) recently identified among murine dermal LECs seemed less clearly defined in our human dermal scRNA-seq data. This could be due to species differences or differences in the immune status of hygienic mice. On the other hand, in a recent study presenting scRNA-seq data from both human and murine nasal lymphatics, *PTX3* appeared to be highly expressed in a subset of LECs in both species (Hong et al., 2023), indicating possible tissue-specific differences. Overall, these findings suggest a more complex immune signature in human skin, likely involving multiple LEC subsets.

Our scRNA-seq dataset identified a small population of capillary LECs (<5% of all LECs, cap2 subset) characterized by reduced expression of *LYVE1* and *CCL21* and a gene expression signature associated with enhanced cell–matrix and focal adhesion. Although the exact function and localization of this subset within lymphatic capillaries remain unclear, we propose that cap2 LECs may exhibit stronger ECM anchoring, due to their increased expression of various integrins and increased assembly of anchoring filaments through upregulation of fibrillin or of the anchoring filament-binding integrin subunits α5 and αv (Bax et al., 2003). Intriguingly, cap2 LECs also displayed upregulation of focal adhesion kinase (*PTK2* or *FAK*) and downstream effectors, including *YAP1* and *TAZ* (*WWTR1*), suggesting a mechano-activated state of this subset. Although alternative explanations are possible, these findings suggest the existence of a specialized capillary LEC subset connected to anchoring filaments and serving as floodgates for increased uptake of lymph under conditions of increased interstitial pressure and edema (Angeli and Lim, 2023; Baluk and McDonald, 2022; Moore and Bertram, 2018; Zawieja, 2009). Intriguingly, the most pronounced gene expression differences between skin and s.c. adipose tissue LECs were observed in the cap1 and cap2 subsets. Pathway analysis further revealed a stronger activation of a mechanoresponsive signature in dermal cap2 LECs, indicating enhanced involvement in focal adhesion, cytoskeletal organization, and migration compared with their counterparts in s.c. adipose tissue (Fig. S2 I). This higher mechanoactivation may reflect differences in ECM composition between the two tissues, or the greater physical strain experienced by the skin as a barrier organ. Given that the cap2/cap1 LEC ratio was also markedly lower in skin compared with the fat (0.15 vs. 0.26, Fig. 3 E), this may suggest increased mechanical tension on cap2 LECs mediated by anchoring filaments in the skin. In the future, newer EM technologies based on 3D volume scanning will likely help to provide further clarity on the distribution of anchoring filaments in human skin (Collinson et al., 2023; Laws et al., 2022).

Our scRNA-seq data revealed the presence of two distinct valve LEC subsets, which based on their differential expression of connexin-37 and connexin-43 (Kanady et al., 2011; Sabine et al., 2012) appeared to correspond to LECs lining the upstream and downstream sides of the valve leaflet. Using 3D confocal imaging, we could unambiguously confirm the expression of select subset-specific genes on the same (CD24 and NEO1) or opposite (CD24 and FOXC2) valve leaflets. Supporting the upstream and downstream identity of these two subsets, we further showed that expression of various flow-responsive genes closely mirrored gene expression patterns observed in human LECs exposed to laminar flow *in vitro*, which mimics shear forces present on the upstream side of the valve leaflet (Angeli and Lim, 2023; Pujari et al., 2020; Sabine et al., 2012; Sabine et al., 2016). Notably, two valve LEC subsets with similar gene expression signatures were also reported in recent scRNA-seq studies of LECs present in human LNs and breast tissue (Abe et al., 2022; Kumar et al., 2023; Takeda et al., 2019). In contrast, so far only one valve LEC subset was reported in scRNA-seq datasets of murine tissues (Gonzalez-Loyola et al., 2021; Petkova et al., 2023), but this could have been due to the overall low number of LECs sequenced.

Our study identified and validated CD24 and NEO1 as new valve-specific markers in human LVs. The valve-specific expression of *Cd24* and *Neo1* was already suggested from previous scRNA-seq studies of murine dermal and mesenteric LECs (Gonzalez-Loyola et al., 2021; Petkova et al., 2023). The preferential expression of both genes in LECs of the upstream sides of the valve leaflets, as shown by 3D confocal imaging, is also supported by *in vitro* experiments demonstrating the flow-induced upregulation of *Cd24* in murine LN LECs and of *NEO1* in primary human dermal LECs. Notably, in contrast to murine LECs, *in vitro* cultured primary human dermal LECs did not express any baseline levels of *CD24*, possibly explaining why we did not find it upregulated by flow in our bulk RNA-seq study (Fig. S5 and Table S3). On the other hand, in flow experiments with murine LECs, *Cd24* was upregulated by shear stress. In line with this, a computational model simulating lymph flow passage through LNs revealed higher laminar shear stress of LECs forming the ceiling of the LN subcapsular sinus (SCS) as compared to LECs forming the SCS floor (Jafarnejad et al., 2015). Intriguingly, in a recent scRNA-seq study, *Cd24* was found to be specifically expressed in SCS ceiling LECs, but not in the other LN LEC subtypes (Fujimoto et al., 2020).

CD24 deficiency caused a modest reduction in mesenteric valve numbers in neonatal mice, whereas no defects were detected in adult skin. A broader assessment of CD24 function across tissues and developmental stages, including lymphatic drainage assays in neonates, will be required to define the functional significance of this phenotype. CD24 promotes cell–cell adhesion through homophilic or heterophilic interactions (Altevogt et al., 2021), as well as cell–ECM adhesion by modulating integrin function (Baumann et al., 2005; Baumann et al., 2012; Runz et al., 2008). Considering that valve LECs are tightly anchored to an ECM core (Shin and Lawson, 2021), CD24 might support valve development by regulating valve LEC adhesion to the ECM and to neighboring valve LECs. However, because CD24 is expressed by multiple cell types (Altevogt et al., 2021; Ayre and Christian, 2016) and our experiments employed global *Cd24* knockout mice, the mesenteric valve phenotype cannot be conclusively ascribed to an LEC-intrinsic defect. Future studies using an LEC-specific *Cd24* knockout will be needed to determine whether *Cd24* directly regulates valve development in LECs. Also, the valve defect observed in the mesentery was much milder than that seen in other valve mutants, such as *Foxc2*- or *Gata2*-deficient mice (Kazenwadel et al., 2015; Petrova et al., 2004). However, it is important to note that certain mutations, such as those in connexin-47 (*GJC2*), can cause severe valve defects and lymphedema in humans, despite having no apparent effect in mice (Ferrell et al., 2010; Meens et al., 2017). To date, mutations in *Cd24* have not been associated with valve defects or lymphedema in either species.

Overall, our study provides a detailed molecular and spatial characterization of human dermal LVs, highlighting not only commonalities but also distinct features of human lymphatic vascular network in comparison with mouse lymphatics. Such knowledge will be important for a better understanding of lymphatic function in human skin homeostasis and pathologies, such as lymphedema.

## Materials and methods

### Mice

C57BL/6, *Prox1-*eGFP (Choi et al., 2011), and *Cd24*^*−/−*^ (Nielsen et al., 1997) mice were bred in our facility. All animals were housed, and experiments were performed under specific pathogen–free conditions. All experiments were approved by the Cantonal Veterinary Office Zurich under Project License ZH040/2022.

### Human tissue sample acquisition and ethical approval

Surplus healthy skin with its underlying s.c. adipose tissue was obtained from routine surgeries from the Department of Plastic Surgery and Hand Surgery of the University Hospital Zurich with the assistance of the SKINTEGRITY.CH biobank. Surplus material from dermolipectomy of the abdomen, arm, or thigh was used for scRNA-seq and immunostainings. Table S1 summarizes the information about the donors whose samples were used for scRNA-seq. Surplus material of healthy skin, resulting from breast reduction, was used for immunostaining. Only biopsies from de-identified donors who gave their written consent were used for further analysis, and donors remained anonymous. The use of human samples for research purposes was approved by the ethics commission of Canton Zurich (KEK 2021-02358 and KEK 2023-00677).

### Antibodies

The details of all primary and secondary antibodies used for this study are provided in Table S4.

### Immunofluorescence staining of skin and adipose tissue sections

Skin or s.c. adipose tissue samples were embedded in optimal cutting temperature compound, snap-frozen on dry ice, sectioned in a cryostat (12–16 µm thick, CryoStar NX50; Thermo Fisher Scientific), and stored at −20°C. Sections were dried at RT for 15 min, and fixed in ice-cold acetone (−20°C) for 2 min at room temperature (RT) and then in 4°C cold methanol for 5 min at RT. After washing the sections three times for 6 min in TBS/0.1% Tween at RT, the slides were dried and blocked for 1 h in ImmunoMix (5% normal donkey serum, 1% bovine serum albumin [BSA], 0.1% Triton X in PBS). Incubation with primary antibodies was performed overnight at 4°C in a humid and dark container. The following day, sections were washed two times for 10 min in PBS at RT on a shaker. Secondary antibodies and Hoechst 33342 (1:5,000, H3570; Invitrogen) were added for 1 h at RT and washed two times for 5 min in PBS at RT on a shaker. Slides were washed once more with TBS/0.1% Tween at RT on a rotator followed by mounting with Mowiol (Sigma-Aldrich). The slides were scanned at 10× magnification with the multispectral slide scanner PhenoImager HT (Vectra Polaris, Akoya) using the Dapi, FITC, Cy3, TxR, and Cy5 filters. Multispectral images of 200× high-power fields were acquired for all slides and were analyzed using the InForm Cell Analysis software (Akoya). The workflow of multispectral image analysis of the vasculature included the following steps done using InForm software: (1) spectral unmixing, (2) tissue segmentation of the LVs and BVs, and (3) measurement of the number and area of LVs or BVs. LVs were identified by positive PDPN expression, and BVs were identified by positive vWF and aSMA expression. Donors 1, 5, and 6 from Table S1 were used for this quantification.

### Preparation of human tissue for confocal whole-mount immunostaining

Confocal imaging was typically performed in skin derived from breast or abdomen. For whole-mount preparation, the tissue was cut into ∼1 × 1 cm pieces and clamped between two microscopy slides with a western blot membrane placed between the bottom of the slide and the sample, epidermis facing up. Using a razor blade, thin slices were cut along the upper microscopy slide (around 150–200 µm depth). Subsequently, circular punch biopsies of 5 or 6 mm diameter were excised using disposable skin biopsy punches (Stiefel/GSK), fixed in 4% paraformaldehyde (PFA) overnight at 4°C, and stored in PBS for up to 6 mo until further use. Punches were blocked overnight in blocking solution (5% BSA, 5% donkey serum, 0.3% Triton X in PBS, or 2% Triton X in PBS for intracellular markers), followed by incubation with primary antibodies in blocking solution for 16–48 h at 4°C. Punches were washed three to four times in 0.3% Triton X in PBS for 30 min each, before incubation with secondary antibodies in blocking solution (24–48 h). Afterward, the washing steps were repeated, and optionally, Hoechst 33342 (1:5,000, H3570; Invitrogen) was added in the second last washing step. Skin punches were mounted in Mowiol (Sigma-Aldrich).

When preparing whole mounts of human s.c. adipose tissue for confocal microscopy, fat pieces of ∼1 × 3 cm were cut into ∼200 µm slices using the same technique described above for the skin. Tissue fixation and staining were also performed according to the same protocol used for the skin. After the last washing step, fat pieces were incubated in RIMS imaging medium (Yang et al., 2014) for 2 days at RT with rotation, changing the medium each day. Fat slices were mounted in RIMS imaging medium.

### Whole-mount immunostaining and quantification of valves in mouse ear skin

Mouse ears were harvested and split along the cartilage, and the dorsal side of the ear was kept and fixed for 30 min in 4% PFA at RT. Subsequently, whole mounts were incubated for at least 2 h with blocking solution (3% BSA in PBS +0.3% Triton X [PBST]). The ears were incubated overnight at RT in primary antibody mix. The next day, the ear skin was washed three times for 5 min in PBST, followed by incubation with secondary antibody mix for 2 h at RT. The ear skin was washed three times for 5 min in PBST before being mounted using Mowiol (Sigma-Aldrich).

For the quantification of dermal valves, ear skin from WT and *Cd24*^−/−^ mice was costained for ITGA9, CD31, and aSMA. CD31^+^ lymphatic capillaries were recognized by morphology. CD31 expression and the presence of intraluminal ITGA9^+^ valves in the presence/absence of LMC coverage were used to divide the lymphatics vasculature into valve-containing pre-collectors (aSMA^−^CD31^+^) and collectors (aSMA^+^CD31^+^). For the quantification of valves, a Tilescan image composed of six individual images was acquired from a central ear skin region comprising predominantly lymphatic pre-collectors or lymphatic collectors (one Tilescan per region and two per mouse). Valves were quantified in each image using LasX software and 3D reconstruction of the tissue.

### Whole-mount immunostaining and quantification of valves in the mouse mesentery

Mesenteries were collected from 4- to 5-day-old littermate pups obtained from *Cd24*^+/−^ × *Cd24*^+/−^ crosses. To allow direct within-litter comparisons, only mesenteries from litters containing at least one +/+ and one −/− pup were included. In those litters, mesenteries from all +/+ and −/− pups were stained and valves quantified. This strategy enabled comparison of precisely age-matched pups, which is otherwise difficult to ensure because the exact time of birth is often unknown (mice are checked only once per day). This is particularly relevant as the analysis was performed at an early developmental stage (4–5 days of age) during which the number of valves still increases rapidly (Sabine et al., 2015). In total, six litters meeting these criteria were analyzed. In two of these litters, more than one pup of a given genotype was present (e.g., one litter contained two +/+ pups and one −/− pup; another contained two +/+ and two −/− pups). Statistical analysis was performed using a linear mixed-effects model with litter included as a random effect, to account for both between- and within-litter variations.

For staining, mesenteries were fixed for 2 h in 2% PFA and subsequently stored in PBS until genotyping results were available. Selected +/+ and −/− mesenteries were pinned as “wheels” on elastomer and processed as previously described (Sabine et al., 2018). Briefly, the mesentery was stretched and flattened to expose vascular branches with mesenteric LN at the center. Small incisions were made at branch–gut and branch–LN junctions to facilitate gentle flushing of vessels with PBS. After washing, mesenteries were fixed with 2% PFA in PBS for 2 h at 4°C, washed with PBS, and blocked/permeabilized with 3% BSA and 0.3% Triton X in PBS. Primary antibodies diluted in blocking solution were applied overnight at 4°C. Following washing with 0.3% Triton X in PBS, samples were incubated with secondary antibodies diluted in blocking solution for 2 h at RT, washed again with 0.3% Triton X in PBS, and finally rinsed with PBS.

For the quantification of valves, fluorescently immunostained mesenteries were imaged in PBS using a Leica M205FA stereomicroscope (PLANAPO 1.0× objective, pE-4000 CoolLED, Prime 95B Photometrics camera, LAS AF6000 software). For each sample, 10–20 images were acquired at 30× magnification across the 488-, 555-, and 647-nm channels with 10–20% overlap. Images were stitched using the Photomerge function of Adobe Photoshop 2024 to reconstruct the entire mesentery. The mesenteric LV network was manually reconstructed based on VEGFR3 staining to determine the total LV length, while lymphatic valves were identified using combinations of VEGFR3 with laminin α5, CD31 or VEGFR2, aSMA, podocalyxin. Total LV length and valve number were quantified in Fiji/ImageJ2 (version 2.14.0; National Institutes of Health [NIH]) using the Skeletonize or Analyse Particle functions, respectively.

### Lymphatic drainage assay in murine ear skin

The lymphatic drainage assay was conducted as described previously (Bachmann et al., 2018; Vranova et al., 2019). Briefly, mice were anesthetized using inhaled isoflurane (2.5%), and the base of the ears was shaved. A volume of 3 μl of the near-infrared dye P20D800 (3 μM, synthesized as previously described [Proulx et al., 2013]) was injected intradermally into the ear using a 29G insulin syringe (Terumo). Mice were then placed in an IVIS imaging system (PerkinElmer), with ears gently taped flat to the isoflurane tubing, and fluorescence images were captured (excitation: 745 nm; emission: 800 nm; exposure: 4 s; binning: small). Imaging was repeated at 1, 2, 4, 6, and 24 h after injection. Between time points, mice were returned to their cages and reanesthetized with isoflurane (∼5 min) for each imaging session.

For analysis, regions of interest (ROIs) were drawn around the ears, and average fluorescence intensity within each ROI was measured using Living Image 4.7.3.20616 software (PerkinElmer). Background fluorescence from uninjected ears was subtracted, and values were normalized to the baseline intensity at time 0. The resulting normalized fluorescence intensities were plotted over time and fitted to a one-phase exponential decay model to calculate the tracer clearance half-life.

### Confocal microscopy and image processing

Confocal images of adult mouse ear skin or mesentery whole mounts and human whole mounts were acquired in Z-stack mode using a Leica TCS SP8 (Leica Microsystems) equipped with eight lasers (405, 458, 477, 488, 496, 514, 561, and 633 nm), a photomultiplier tube (PMT) detector, and two Leica hybrid detectors using the following objectives: 10×/0.3 (PH1 HC PL FLUOTAR), 20×/0.7 (PH2 HC PLAN APO), 40×/1.1 Water (HC PL IR APO CORR). In the case of the images generated for the quantification of valves in WT and *Cd24*^−/−^ ear skin, a Leica Stellaris SP8 DIVE with White Light Laser and five internal Power HyD detectors using 20×/0.75 (HC PL APO CS2) was used. Images were acquired at RT using the Leica LASX SP8 or Leica LASX Stellaris 8 software. Each image represents the maximum intensity projection of a Z-stack of single tiles or a Tilescan generated from six images. All images were processed and generated using Fiji/ImageJ2 software (version 2.14.0; NIH) or Imaris x64 software (version 10.2.0; Bitplane). For Imaris, microscopy files were first converted to Imaris files (.ims) using ImarisFileConverter and 3D pictures were created using the “snapshot” tool.

### iDISCO clearing protocol for light-sheet microscopy performed on human skin and s.c. adipose tissue samples

Human skin and attached s.c. adipose tissue were separated using surgical scissors. In the case of the skin, circular punch biopsies of 3–5 mm diameter (thickness ∼1–5 mm) were excised using disposable skin biopsy punches (Stiefel/GSK). S.c. adipose tissue was cut into 0.3–0.4 mm pieces using surgical scissors. Samples were subsequently fixed in 4% PFA overnight at 4°C and were clarified using a modified iDISCO^+^ protocol (Kajiya et al., 2018) as described below. Samples were washed in PBS/0.2% Tween-20 at RT before transferring them into a blocking/permeabilization solution consisting of 3% BSA, 10% (wt/vol) Triton X, and 10% (wt/vol) N-butyldiethanolamine in H_2_O for 1 wk to hyperhydrate the samples for improving antibody penetration (Bauer et al., 2024). Alternatively, samples were blocked and permeabilized in a solution consisting of 1% BSA, 0.2% Triton X, 20% DMSO, 0.02% NaN_3_, 2% glycine in PBS for 2 days followed by 1% BSA, 0.2% Triton X, 6% donkey serum, 10% DMSO, 0.02% NaN_3_ in PBS for 3 days. The blocking/permeabilization step was carried out at 4°C with gentle shaking, refreshing the reagent daily. Following this, samples were washed in PBS/0.2% Tween-20 for 24 h at RT. Next, samples were incubated with primary and secondary antibodies diluted in PermBlock solution (3% [wt/vol] BSA, PBS/0.1% Tween-20) at 4°C for 1 wk each. In between primary and secondary antibodies, samples were washed in PBS/0.2% Tween-20 at least four times for a minimum of 1 h per wash. The final wash was performed in PBS/0.2% Tween 20 overnight prior to clearing. For the clearing process, samples underwent two consecutive incubations in dichloromethane (DCM, #270997-12X100ML; Sigma-Aldrich) for 15 min each, until the samples sank. Subsequently, the samples were immersed in dibenzyl ether (DBE, #108014-1KG; Sigma-Aldrich) for refractive index matching, preparing them for light-sheet imaging.

### Light-sheet 3D imaging and image processing

3D imaging was performed using light-sheet microscopy with an Ultramicroscope Blaze (Miltenyi Biotec) controlled by InspectorPro software (Miltenyi Biotec). Samples were placed in an imaging reservoir filled with DBE and illuminated from the side by a laser light sheet. The light sheet was generated by a laser with wavelengths of 488, 561, or 640 nm. Imaging was conducted using a 4× objective at various magnifications (1×, 1.66×, and 2.5×), with the thickness of the light sheet set to 4 μm. A 4.2 Megapixel sCMOS camera (2,048 × 2,048 pixel size) captured the images, with a step size between each image fixed at 6 μm. Alternatively, samples were imaged using the mesoSPIM light-sheet microscope (mesoSPIM Benchtop) controlled by an open-source software based on Python and PyQt5, as described in reference (Vladimirov et al., 2023, *Preprint*; Voigt et al., 2019). For imaging, the embedded samples were mounted in a quartz cuvette filled with index-matching DBE. This cuvette was then placed in an immersion chamber (Portmann Instruments UQ-753; dimensions: 40 × 40 × 50 mm), which was also filled with DBE. Light-sheet illumination was provided sequentially from the left side using 488-, 594-, and 647-nm lasers. Imaging was conducted using Mitutoyo M Plan Apo 7.5× or 5× objectives. A sCMOS camera captured the images (Photometrics Iris 15), with a z-step size of 10 μm. Alternatively, 3D light-sheet imaging was performed using the ZEISS Lightsheet 7 (ZEISS) with multi-positioning imaging (XYZ movement and rotation) equipped with the ZEN (black edition) 3.1 LS (ZEISS). On the day before imaging, the samples were transferred from DBE into ethyl cinnamate (ECi) to ensure medium exchange. For imaging, samples were glued into a mounting stage and submerged into an imaging chamber containing ECi. The refractive index of detection and illumination optics was adjusted accordingly. The samples were illuminated using dual illumination generating a light sheet with the wavelengths 488, 561, and 638 nm, which originated from 10×/0.2 illumination optics. Images were acquired using a 5×/0.16 dry objective with various magnifications (0.6×–1.00×) and a pixel size of 1,920 × 1,920. Images were captured using a dual camera detection module with two PCO.edge sCMOS cameras, with the optimal step size as suggested by the software. Correction of chromatic aberration in the acquired images was achieved through the TransformJ Translate package of Fiji/ImageJ2 software (version 2.16.0; NIH).

Images, 3D volumes, and movies were generated using Imaris x64 software (version 10.2.0; Bitplane). Z-stack light-sheet images were first converted to Imaris files (.ims) using ImarisFileConverter. 3D pictures and movies were created using the snapshot and “animation” tools.

### Quantification of LEC morphology around valves

Valves in confocal images of upper human dermis or light-sheet images of full-thickness human skin were identified using VE-cadherin in combination with PDPN staining to distinguish LVs from BVs. For the evaluation of LEC morphology, a circle with a radius of 60 μm was drawn around the center of each valve and the LECs in the upstream and downstream part of the circle were assessed based on their morphology as either “oak leaf”–shaped, “cuboidal/mixed”–shaped, or “elongated.” In case no assessment was possible (e.g., due to bad image quality), the region was excluded from the analysis. The assessment of LEC morphology was performed by three independent experts. For each of them, the percentage of LEC morphological states among all valve regions evaluated was determined. Subsequently, the average of the three independent assessments was determined and plotted. Overall, 50 valve regions from a total of 28 valves from six donors were analyzed (27 light-sheet and 23 confocal imaging).

### Culture of single donor human dermal LECs

Juvenile single donor male human dermal LECs were purchased from PromoCell (C-12216, lot numbers: 431Z006.2, 439Z007.2, and 433Z032.3). Cells were grown on human plasma fibronectin (10 µg/ml, FC010; Millipore)–coated dishes in complete EBM-2 medium (CC-3156; Lonza, 5% FBS including VEGF-A). Cells were used in bulk RNA-seq experiments between passages 3 and 6.

### Culture of immortalized murine LECs

Conditionally murine imLECs expressing a heat-labile version of the large T antigen (Vigl et al., 2011) were cultured in media containing 40% DMEM (low glucose), 40% F12-Ham, 20% FBS, 1× antibiotic–antimycotic solution (all from Gibco), 56 µg/ml heparin (H3149; Sigma-Aldrich), 10 µg/ml EC growth supplement (211-GS; AbD Serotec), and 2 nM L-glutamine (25030-024; Invitrogen). Dishes were coated with 10 µg/ml collagen type I (5005-B; Advanced BioMatrix) and 10 µg/ml fibronectin (FC010; Millipore). For expansion, imLECs were cultured at 33°C in media supplemented with 1 U/ml murine interferon-gamma (IFNγ, 313-05; PeproTech) to induce large T antigen expression (Vigl et al., 2011). Around 48–72 h prior to the assay, imLECs were seeded and grown to confluency at 37°C in media without IFNγ.

### Isolation and culture of LN LECs

Primary LN LECs were isolated and cultured as previously described (Russo et al., 2021). In brief, skin-draining LNs (popliteal, inguinal, axillary, brachial, and auricular) were isolated from WT mice and digested in RPMI medium supplemented with 0.25 mg/ml Liberase DH (05-401-054-001; Roche) and 200 U/ml DNase I (11284932001; Sigma-Aldrich) for 1 h at 37°C. After digestion, the cell suspensions were filtered through 70 µm cell strainers and cultured on cell culture dishes pre-coated with 10 µg/ml collagen type I (5005-B; Advanced BioMatrix) and 10 µg/ml fibronectin (FC010; Millipore) in LN LEC media (Minimal Essential Medium-alpha medium containing 10% FBS and 1× penicillin/streptomycin [all from Gibco]). Upon reaching >80% confluency (days 5–7), plates were a mixture of LN stromal cells, fibroblastic reticular cells, and LECs. Cells were detached with Accutase for 5 min at 37°C, washed, and purified using CD31^+^ microbeads (130-097-418; Miltenyi Biotech). Isolated LECs were seeded on collagen and fibronectin-coated cell culture dishes and kept for up to six passages after isolation.

### Staining of *in vitro* cultured cells for flow cytometry

Single-cell suspensions of *in vitro* cultured cells were prepared in U-bottom 96-well plates. Cells were incubated first with Fc receptor blocking solution and live-dead Zombie NIR (1:500, 423106; BioLegend) or eFluor 780 fixable viability dye (1:1,000, 65-0865-14; eBioscience) in PBS for 10 min at 4°C. Primary antibodies diluted in FACS buffer (PBS buffer, 2.5% FBS, 2 mM EDTA) were added to each well for 15 min at 4°C. After washing once with 200 μl of FACS buffer, cells were incubated with secondary antibodies (if needed) for 15 min at 4°C, followed by one last wash. Data were acquired on a CytoFlex S flow cytometer (Beckman Coulter) equipped with four lasers (405, 488, 561, 633 nm) or two lasers (488, 633 nm) and analyzed using FlowJo software version 10.8.1 (BD Life Sciences).

### *In vitro* flow assay

The parallel plate system (10902; Ibidi) was used to create isolated laminar and oscillatory shear stress conditions. Ibidi slides (µ-slides I^0.8^ Luer slides, 80196; Ibidi) were coated with the appropriate coating solution depending on the cell type for 30 min at RT. Subsequently, the coating was removed, and the cells were seeded on the slides at a density of 200,000 cells per slide and cultured for 24 h at 37°C. The cells were subjected to laminar flow (4 dyn/cm^2^) and oscillatory flow (4 dyn/cm^2^; 0.25 Hz, flow changes direction every 4 s), or kept under static conditions for 48 h. The red tubing set (10962; Ibidi) was used. The medium was renewed on the day of shear stress induction and after 24 h in the case of the static condition. For RNA isolation, the medium was aspirated and 300 μl lysis buffer (740955.50; NucleoSpin kit, Macherey-Nagel) was added to the slides and collected.

### RNA isolation, conversion to cDNA, and gene expression analysis by quantitative PCR

Total RNA was isolated using the NucleoSpin kit (740955.50; Macherey-Nagel) following the manufacturer’s protocol (including the DNase treatment) and quantified by NanoDrop (NanoDrop One, Thermo Fisher Scientific). Total RNA was reverse-transcribed using High-Capacity cDNA Reverse Transcription Kit (Applied Biosystems) according to the manufacturer’s instructions.

Quantitative PCR was performed on reversely transcribed RNA using PowerUp SYBR Green Master Mix (A-25776; Thermo Fisher Scientific). Gene expression analysis was conducted using the following primers (Microsynth): *18s* (forward 5′-AGG​AAT​TCC​CAG​TAA​GTG​CG-3′, reverse 5′-GCC​TCA​CTA​AAC​CAT​CCA​A-3′); mouse *Klf4* (forward 5′-CGA​CTA​ACC​GTT​GGC​GTG​A-3′, reverse 5′-GAG​GTC​GTT​GAA​CTC​CTC​GG-3′), mouse *Cd24* (forward 5′-ACA​TCT​GTT​GCA​CCG​TTT​CCC​G-3′, reverse 5′-CAG​GAG​ACC​AGC​TGT​GGA​CTG-3′), human *CCL21* (forward 5′-AGC​AGG​AAC​CAA​GCT​TAG​GCT​G-3′, reverse 5′-GGT​GTC​TTG​TCC​AGA​TGC​TGC​A-3′), human *LYVE1* (forward 5′-GCC​GAC​AGT​TTG​CAG​CCT​ATT​G-3′, reverse 5′-CCG​AGT​AGG​TAC​TGT​CAC​TGA​C-3′) and acquired on a QuantStudio 7 Flex system (Applied Biosciences).

### *In vitro* cycling stretch of human dermal LECs

Polydimethylsiloxane (PDMS) membranes of 390 ± 10 µm thickness were fabricated, and 14-mm-diameter punches were prepared as previously reported (Bachmann et al., 2016; Bernardi et al., 2017). Before use, membranes were treated with plasma (100 W for 30 s, 1 ± 0.2 mbar) in 24-well plates to increase their hydrophilicity and then coated with 10 µg/ml fibronectin (FC010; Millipore) in PBS to promote cell adhesion. Approximately 50,000–75,000 cells per well were seeded and monolayers left to mature for 72 h. An inflation-based custom-made bioreactor (Bachmann et al., 2016; Wu et al., 2024) was then used to apply 320 mbar internal pressure leading to an equibiaxial tension state in the central part of the PDMS substrate. The corresponding deformation of the endothelial monolayers was ∼10% (apex principal strain). The strain cycles were applied at a frequency of 0.03 Hz for 18 h. At the endpoint, samples were either fixed with 4% PFA and processed for immunofluorescence or lysed with 250 μl RNA lysis buffer (740955.50; NucleoSpin kit, Macherey-Nagel) and processed for RT-qPCR.

### Immunofluorescence and imaging of stretched human dermal LECs

Fixed membranes were permeabilized for 10 min with 0.5% Triton X in PBS and incubated for 1 h at RT in a blocking solution of 2% BSA in PBS. Samples were then incubated with rabbit anti-mouse VE-cadherin (#2500, clone D87F2, 1:200; Cell Signaling) diluted in blocking solution overnight at 4°C, and washed with PBS, followed by incubation with secondary antibody chicken anti-mouse IgG-AF647 (A21463, 1:250; Thermo Fisher Scientific) and DAPI (62248, 1:1,000; Thermo Fisher Scientific) for 1 h at RT. Image acquisition was performed at RT using an automated Nikon-Ti spinning disk confocal microscope (Nikon) equipped with an Andor DU-888 camera (Oxford Instruments) and a pE100 LED illumination system (CoolLED Ltd). For comparison purposes, different sample images of the same antigen were acquired under constant acquisition settings. Image acquisition was performed using a 20×/0.75 NA air objective (Plan Fluor, Nikon). Cell elongation was obtained from the VE-cadherin signal. The cell outline was derived using the “polygon selections” tool of Fiji/ImageJ (NIH) software. It was then postprocessed by MATLAB to measure the aspect ratio (A.R.) of individual cells by fitting an ellipsoid to the cell outline. The A.R. was calculated as the ratio between the long axis and the short axis of the fitted ellipse.

### Isolation of the stromal vascular fraction from human skin and s.c. adipose tissue

Human tissue samples (∼3–15 cm^2^ of skin and underlying fat of up to 3–5 cm height) were washed with Hank’s Balanced Salt Solution supplemented with 5% FBS, 2% antibiotic–antimycotic solution (Gibco), and 20 mM HEPES (all from Gibco) before separating the skin from the s.c. adipose tissue using surgical scissors. Both tissues were individually minced and digested enzymatically in 1,000 U/ml collagenase type I (LS004197; Worthington) and 40 µg/ml DNase I (11284932001; Roche) in RPMI 1640/GlutaMAX medium supplemented with 10% FBS and 1% antibiotic–antimycotic solution (all from Gibco) for 10 min in a 37°C water bath followed by 1 h at 37°C under constant agitation. Digested tissues were separately smashed with a plunger and filtered through a 100-µm cell strainer, further washed with supplemented RPMI medium, and centrifuged at 300 *g* for 8 min. The suspension was passed again through a 100-µm cell strainer. The resulting stromal vascular fraction was cryopreserved in a total of 1 ml of 90% FBS and 10% DMSO (long-term storage in liquid nitrogen).

### Staining of the stromal vascular fraction derived from human tissue for flow cytometry

Single frozen vials of the stromal vascular fraction were thawed at 37°C in a water bath, and the content was transferred to 9 ml of RPMI 1640 GlutaMAX medium supplemented with 10% FBS and 1% antibiotic–antimycotic solution (all from Gibco) before centrifugation at 300 *g* for 5 min. Cells were incubated first with human Fc receptor blocking solution (1:10, 422302; BioLegend) and eFluor 780 fixable viability dye (1:1,000, 65-0865-14; eBioscience) in PBS for 5 min at 4°C. Afterward, the cell suspension was transferred to a 5-ml round-bottom tube and the antibodies (CD45-BV421, CD31-PE, PDPN-Pe/Cy7 for sorting of LECs) were directly added to the cell suspension and left to incubate for 15 min at 4°C. For the scRNA-seq experiment, hashtag antibodies (see Table S4) were added at the same time as the primary antibodies. TotalSeq-B anti-human hashtag antibodies (used at a dilution of 1:25) recognizing CD298 and β2 microglobulin (which are expressed on virtually all human cells, including LECs) were used to label individual samples with a specific oligonucleotide. Cell suspensions were washed two times with 6 ml FACS sorting buffer (Ca/Mg^++^-free PBS buffer and 1% FBS) and centrifuged at 300 *g* for 5 min. Cell suspensions were filtered through a 40-µm filter right before cell sorting or acquisition on a CytoFlex S flow cytometer (Beckman Coulter) equipped with four lasers (405, 488, 561, and 633 nm) and CytoFlex S instrument (Beckman Coulter). Data were analyzed using FlowJo software version 10.8.1 (BD Life Sciences).

### Single-cell transcriptomics of human LECs

Sorting of LECs (CD45^+^CD31^+^PDPN^+^) was performed on a BD Aria Fusion Cell Sorter equipped with five lasers (355, 405, 488, 561, and 633 nm) using a 100-µm nozzle. Sorted cells were collected in a 1.5-ml tube containing FACS sorting buffer at 4°C. After sorting, the 1.5-ml tube containing the sorted cells was centrifuged at 300 *g* for 5 min and the supernatant was removed until ∼55 μl was left. The quality and quantity of the single-cell suspension were evaluated with a hemocytometer under a Leica DM IL LED Fluo microscope. Approximately 5,000–20,000 cells per sample pool (each donor and cell type were labeled with different TotalSeq-B hashtag antibodies) were loaded into the 10X Chromium controller. The library preparation followed the manufacturer’s guidelines specified in the Chromium Single Cell 3′ Reagent Kits User Guide (version 3.1 Chemistry Dual Index) with Feature Barcoding technology for Cell Surface Protein. For sequencing, the resulting libraries were processed on an Illumina NovaSeq 6000 SP Flow Cell. The sequencing parameters were set according to 10X Genomics recommendations, using paired-end reads with the following specifications: R1 = 28, i7 = 10, i5 = 10, R2 = 90. An average depth of around 50,000 reads per cell was achieved during sequencing. CellRanger (version 7.1.0) mkfastq (Zheng et al., 2017) was used to generate demultiplexed gzipped fastq files from the Illumina NovaSeq raw sequencing data. CellRangerMulti (version 7.1.0) was subsequently used for read alignment to the human reference genome (build GRCh38.p13), barcode processing, and unique molecular identifier counting using gene model GENCODE Release 42.

### scRNA-seq data processing

Quality control and preprocessing were performed using R (version 4.3.2) and the R/Bioconductor package scater (version 1.28.0 [McCarthy et al., 2017]). The sorted cells of low quality were filtered and excluded from further analysis based on the high expression of mitochondrial genes (>30%), a low number of expressed genes (<200 detected genes), and low or high overall unique molecular identifier counts (>4 median absolute deviation from the median across all cells of the same sample). Contaminating cells such as BECs, fibroblasts, keratinocytes, smooth muscle cells, and leukocytes were identified based on the expression of contaminant markers (*ACTA2*, *COL1A1*, *FLT1*, *JAM2*, *KRT14*, *KRT5*, *PDGFRB*, *PTPRC*) and excluded from further analysis, resulting in a final dataset of 21,374 cells (fat: 7,300; skin: 9,369; mixed: 4,705) after quality control. Gene selection and integration of data from different donors (Fig. S3, C and D) were performed in Python (version 3.10.12) using the scanpy module (version 1.9.6 [Wolf et al., 2018]). For each donor, the top 500 highly variable genes (HVGs) were selected based on the ranked Pearson residual variance of a negative binomial offset model as described in Lause et al. (2021). HVGs shared by at least two donors, as well as known marker genes (*PROX1*, *PDPN*, *LYVE1*, *CCL21*, *RELN*, *ACKR4*, *CCL2*, *CXCL2*, *CLDN11*, *CD24*, *ESAM*, *ALCAM*, *ICAM1*, *PIEZO1*, *PIEZO2*) resulting in a gene set of 675 genes, were used for batch integration and subsequent clustering. Log-normalized and scaled gene expression counts were used for principal component analysis (PCA), and the first 100 PCs were used to integrate cells from different donors using harmonypy (version 0.0.9 [Korsunsky et al., 2019]).

### Comparative analysis between subsets and tissue types

Relevant cell populations within the integrated single-cell data were identified using graph-based clustering based on the Leiden algorithm (Traag et al., 2019) at different resolutions. Cluster marker genes were determined using the area under the curve (AUC) of gene-specific pairwise cluster comparisons. Genes with the smallest rank across all pairwise comparisons were selected as implemented in the scoreMarker function of the R/Bioconductor scran package (version 1.28.2 [Lun et al., 2016]). Clusters were annotated based on the top-ranked marker genes and the expression of known marker genes of major lymphatic cell types, such as capillary (*LYVE1*^*++*^*CCL21*^*++*^*),* pre-collector (*LYVE1*^*+*^*CCL21*^*+*^), collector (*LYVE1*^*−*^*CCL21*^*−*^*ACKR4*^*+*^), valve (*CLDN11*^*+*^, *CD24*^*+*^), and proliferative (*MKI67*^*+*^, *AURKB*^*+*^) LECs. Cell type–specific DEGs between different tissue types (adipose tissue, skin) were obtained by pseudobulk DE analysis adjusting for patient-specific differences using the Bioconductor/R edgeR package (version 3.42.4 [Robinson et al., 2010]). Similarly, differential abundance of cell types between tissue types was obtained using the Bioconductor/R edgeR package (version 3.42.4 [Robinson et al., 2010]) accounting for patient-specific differences. Cells from a donor with only one tissue type acquired were excluded from differential abundance and DE analysis.

### Pathway analysis

For enrichment analysis, we used Enrichr and filtered significant pathways by applying adjusted P value <0.05 (Chen et al., 2013; Kuleshov et al., 2016; Xie et al., 2021). The selected top pathways are shown in the figures. For the capillary two enrichment analysis, marker genes were ranked according to the AUC and the top 50 marker genes were selected (Data S4). For the upstream and downstream valve subset analysis, the top 50 upregulated DEGs from each valve subset were selected (Data S5).

### Bulk sequencing of human dermal LECs subjected to shear stress

Human dermal LECs (passages 3–6) were subjected to laminar (4 dyn/cm^2^) or oscillatory (4 dyn/cm^2^, 0.25 Hz) shear stress using the parallel plate system described in the relevant section. Total RNA was isolated using the NucleoSpin kit (740955.50; Macherey-Nagel) and stored at −80°C until further processing. RNA concentration and purity determination (Agilent TapeStation 2200), library preparation (TruSeq Stranded mRNA; Illumina), and sequencing (Illumina NovaSeq 6000) were performed by the Functional Genomics Center Zurich (FGCZ). Reported RNA integrity numbers were all above 9.3, and sequencing depth was at least 22.9 million reads per sample. Sequencing reads were mapped to the human reference genome (GRCh38) and aligned using the STAR package, counted using the Rsubread package, and differential expression analysis was conducted using the DESeq2 package (with grouping according to treatment and donor), done by the FGCZ, all in R version 4.0.4.

Subsequent data analysis and visualization: PCA was conducted using the DESeq2 package, visualized with ggplot2. Heatmaps were visualized with the R library “ComplexHeatmap.” Clusters of deferentially expressed genes were determined by the pam function from the “cluster” R package, in R version 4.0.5. Based on the gap statistics (Tibshirani et al., 2001) method from the R package “factoextra,” three clusters were selected for subsequent pathway enrichment analysis by g:Profiler (Reimand et al., 2007).

### Comparative analysis of capillary subsets and valve subclusters

To compare the cap1 and cap2 subsets, the gene signatures were determined using pseudobulk DE analysis between cap1 and cap2 subsets adjusting for patient-specific differences using the Bioconductor/R edgeR package (version 3.42.4 [Robinson et al., 2010]). To characterize valve subcluster gene signatures, gene expression profiles were compared with bulk RNA-seq data from human dermal LECs subjected to static conditions, or laminar or oscillatory shear stress. Gene signatures of valve subclusters were determined using pseudobulk DE analysis between valve subcluster adjusting for patient-specific differences using the Bioconductor/R edgeR package (version 3.42.4 [Robinson et al., 2010]). Based on the top DEGs, correlation coefficients between each bulk RNA-seq sample and valve subcluster were calculated using the clustify function of the Bioconductor/R package clustifyr (version 1.12.0 [Fu et al., 2020]). The similarity of valve subclusters and gene expression patterns associated with different flow conditions in bulk RNA-seq samples were further analyzed based on the expression of the top DEGs within valve subclusters. Vice versa, the overlap of DEGs between flow conditions in the bulk RNA-seq data and DEGs of the valve subclusters was determined.

### Statistical analysis

Results are presented as the mean ± standard deviation. GraphPad Prism 10 was used for graphic representation and statistical analysis of the data. A normality test was used to assess whether the data between two groups were normally distributed. For normally distributed data, a parametric unpaired or paired two-tailed Student’s *t* test was used. When the data were not normally distributed, a nonparametric Mann–Whitney test was used instead. The figure legend contains information about the statistical test used. For valve analysis in the mouse mesentery, data were analyzed using a linear mixed-effects model with the genotype (WT vs. KO) as a fixed effect and breeding as a random effect to account for variability between litters. The analysis was performed in R using the lmer() function from the *lme4* package, allowing to test for genotype differences while appropriately modeling the non-independence of animals derived from the same breeding. Differences were considered statistically significant when P < 0.05.

### Online supplemental material

Fig. S1 shows the quantification of LVs in 2D sections of human skin and adipose tissue. Fig. S2 shows supplemental data for the scRNA-seq and data comparing the gene expression between human skin and adipose tissue. Fig. S3 shows the expression of various genes in the seven subsets identified in the scRNA-seq. Fig. S4 shows the data related to the bulk RNA-seq of human dermal LECs subjected to laminar or oscillatory shear stress. Fig. S5 shows CD24 expression in cultured LECs and in murine ear skin, as well as the quantification of valve numbers and lymphatic drainage function in the ear skin of WT and *Cd24*^−/−^ mice. Table S1 provides information on the human tissue donors and LEC counts by subset and tissue achieved upon FACS-based LEC isolation and scRNA-seq. Table S2 shows the top 20 cluster marker genes for each LEC subset. Table S3 shows the flow-dependent gene expression changes in primary dermal LECs cultured *in vitro*, highlighting key marker genes associated with the identified two valve LEC subclusters. Table S4 shows a combined list of all antibodies used for immunofluorescence, flow cytometry, and hashtagging performed for scRNA-seq. Data S1 shows the top 100 DEGs between LECs on the upstream or downstream sides of the valve leaflets sorted by adjusted P value. Positive logFC represents gene DE in LECs on the downstream sides of the valve leaflets, whereas negative logFC represents gene DE in LECs on the upstream sides of the valve (FC, fold change). Data S2 shows the top 50 most significant upregulated or downregulated genes in human dermal LECs subjected to laminar shear stress (4 dyn/cm^2^) compared with static conditions (bulk RNA-seq dataset). Genes with a positive log_2_ ratio are upregulated under laminar shear stress, whereas genes with a negative log_2_ ratio are downregulated under laminar shear stress. Data S3 shows the top 50 most significant upregulated or downregulated genes in human dermal LECs subjected to oscillatory shear stress (4 dyn/cm^2^, 0.25 Hz) compared with static conditions (bulk RNA-seq dataset). Genes with a positive log_2_ ratio are upregulated under oscillatory shear stress, whereas genes with a negative log_2_ ratio are downregulated under oscillatory shear stress. Data S4 shows the list of cap2 marker genes used for enrichment analysis. Data S5 shows the list of DEGs in upstream and downstream valve LECs used for enrichment analysis. Video 1 shows LYVE-1^+^ LVs in green and aSMA^+^ vessels in red in a punch biopsy of human breast skin imaged by light-sheet microscopy. Video 2 shows PDPN^+^ LVs in green and aSMA^+^ vessels in red in a punch biopsy of human thigh skin imaged by light-sheet microscopy. Video 3 shows vWF^+^ BVs in green and aSMA^+^ vessels in red in a punch biopsy of human breast skin imaged by light-sheet microscopy.

## Supplementary Material

Table S1 shows an overview of human LEC sequencing (donor details and subset counts).

Table S2 shows top 20 cluster marker genes for each LEC subset.

Table S3 shows flow-dependent transcriptional profiles of primary dermal LECs.

Table S4 lists antibodies.

Data S1 shows the top 100 DEGs between LECs on the upstream or downstream sides of the valve leaflets sorted by adjusted P value.

Data S2 shows the top 50 most significant upregulated or downregulated genes in human dermal LECs subjected to laminar shear stress (4 dyn/cm^2^) compared with static conditions (bulk RNA-seq dataset).

Data S3 shows the top 50 most significant upregulated or downregulated genes in human dermal LECs subjected to oscillatory shear stress (4 dyn/cm^2^, 0.25 Hz) compared with static conditions (bulk RNA-seq dataset).

Data S4 shows the list of cap2 marker genes used for enrichment analysis.

Data S5 shows the list of DEGs in upstream and downstream valve LECs used for enrichment analysis.

## Data Availability

Raw data has been made available in the ETH research collection (public repository) (URL: https://www.research-collection.ethz.ch; DOI: https://doi.org/10.3929/ethz-c-000783898). The scRNA-seq, R file containing raw counts, and metadata and bulk RNA-seq data generated for this publication have been deposited in NCBI’s Gene Expression Omnibus (GEO) and are accessible through the GEO Series accession number GSE282417. Preprocessed scRNA-seq data are available for exploration at https://halinethz.shinyapps.io/shinyapp/. All the scRNA-seq analyses are available on GitHub (https://almutlue.github.io/lec_flow_push/about.html and https://github.com/almutlue/lec_flow_push/tree/main).
