# Supplementary material for: Transcriptomics- and 3D imaging–based characterization of the lymphatic vasculature in human skin
Source: J Exp Med. 2025 Nov 4;223(1):e20242353. doi: 10.1084/jem.20242353 (PMC12584878; doi:10.1084/jem.20242353)
Supplement: Table S1 — shows an overview of human LEC sequencing (donor details and subset counts). [file jem_20242353_tables1.docx]

**Table S1. Overview of Human LEC sequencing: Donor details and subset counts.**

| **Information about the human donors used for the scRNA-seq and the number of LECs sequenced (counts after pre-processing of the data)** | | | | | | |
| --- | --- | --- | --- | --- | --- | --- |
| **Subjects for scRNA-seq** | | | | **Number of LECs sequenced** | | |
| **Subject** | **Sex** | **Year of Birth** | **Location** | **Total LECs** | **Skin-derived LECs** | **Adipose tissue-derived LECs** |
|  |  |  |  |  |  |  |
| 1 | F | 1979 | Abdomen | 3902 | 3744 | 158 |
| 2 | F | 1982 | Arm | 2773 | 0 | 2773 |
| 3 | F | 1982 | Abdomen | 1247 | 539 | 708 |
| 4 | F | 1973 | Abdomen | 631 | 631 | 0 |
| 5 | M | 1958 | Abdomen | 4705 | n.a.* | n.a.* |
| 6 | M | 1988 | Abdomen | 3647 | 1681 | 1966 |
| 7 | F | 1987 | Thigh | 4469 | 2822 | 1647 |
| ***Total*** | | | | **21374** | **9417** | **7252** |
|  | | | | | | |
| **Number of LECs sequenced per subset and tissue (post pre-processing)** | | | | | | |
| **LEC Cluster** | | | | **Skin-derived LECs** | **Adipose tissue-derived LECs** | **LECs of mixed origin*** |
| Capillary 1 | | | | 3108 | 1748 | 1167 |
| Capillary 2 | | | | 453 | 458 | 245 |
| Pre-collector 1 | | | | 2494 | 1750 | 1163 |
| Pre-collector 2 | | | | 2292 | 2249 | 1500 |
| Collector | | | | 243 | 613 | 249 |
| Valve | | | | 735 | 449 | 371 |
| Proliferative | | | | 44 | 33 | 10 |
| ***Total*** *(total of 21,374)* | | | | **9369** | **7300** | **4705** |

*: Due to a technical issue with the hashtags, LECs from donor 5 could not be assigned to their tissue of origin (skin or adipose tissue) and were instead retrieved as “mixed origin” by selecting unhashed barcodes. Consequently, these LECs were only included in analyses combining LECs from both tissues (e.g., Fig. 2 F).
