# Supplementary material for: Transcriptomics- and 3D imaging–based characterization of the lymphatic vasculature in human skin
Source: J Exp Med. 2025 Nov 4;223(1):e20242353. doi: 10.1084/jem.20242353 (PMC12584878; doi:10.1084/jem.20242353)
Supplement: Table S2 — shows top 20 cluster marker genes for each LEC subset. [file jem_20242353_tables2.docx]

**Table S2. Top 20 cluster marker genes for each LEC subset. Cells in each cluster were compared with all cells from the other clusters.**

| **Cluster** | **Top 20 cluster markers** |
| --- | --- |
| **Capillary 1** | TXNIP; GNG11; CCL21; RPL41; PLCG2; TFPI; MMRN1; RPL34; ATP5F1E; S100A10; LYVE1; PPFIBP1; RPL10; RPS8; PKHD1L1; MAF; TFF3; RPL9; RPL26; TM4SF1 |
| **Capillary 2** | MAGI1; KALRN; TSHZ2; AUTS2; PKHD1L1; STOX2; JMJD1C; PPFIBP1; UTRN; FNDC3B; CD36; DOCK5; LRMDA; MACF1; TRIO; ZFPM2; ARHGAP26; ELMO1; ZBTB20; ASAP1 |
| **Pre-collector 1** | MMRN1; CNKSR3; LYVE1; RPL41; NFKBIA; SPAG9; FNDC3B; IRF1; CCL21; SIK3; TPT1; RPL34; PPFIBP1; JUNB; TSHZ2; TM4SF1; KLF6; RHOJ; DENND4A; GADD45B |
| **Pre-collector 2** | RPL34; CCL21; RPL41; NFKBIA; RPL32; GADD45B; TFF3; RPL10; S100A10; RPS12; CEBPD; JUNB; RPS8; TM4SF1; EIF1; ATP5F1E; RPL39; GNG11; ID3; FABP5 |
| **Collector** | CXCL2; CLU; NFKBIA; RPLP1; SERPINB1; NNMT; RPL41; NTS; TPT1; MT2A; RPL34; IL33; CCL2; TM4SF1; CDKN1A; CEBPD; RPL10; RPS8; THBD; LDB2 |
| **Valve** | PTMA; ARL15; SOX4; CALM1; TMSB10; B2M; SLC41A1; CD24; RPL41; ALCAM; RPL34; DNAJA1; PDE4D; CALD1; GADD45B; RPS28; PROX1; NFKBIA; RPS8; PLPP1 |
| **Proliferative** | LYVE1; TUBA1B; RPLP1; TPM4; LGALS1; CCL21; MKI67; RPL41; S100A10; TM4SF1; H2AZ1; GNG11; FAM25G; PFN1; TMSB10; HMGB2; UBE2S; ATP5F1E; RPL10; MMRN1 |
