# Supplementary material for: Transcriptomics- and 3D imaging–based characterization of the lymphatic vasculature in human skin
Source: J Exp Med. 2025 Nov 4;223(1):e20242353. doi: 10.1084/jem.20242353 (PMC12584878; doi:10.1084/jem.20242353)
Supplement: Table S3 — shows flow-dependent transcriptional profiles of primary dermal LECs. [file jem_20242353_tables3.docx]

**Table S3: Flow-dependent transcriptional profiles of primary dermal LECs.**

| **subcluster 1 (upper valve)** | **laminar vs static (fold change)** | **3 out of 3 experiments** | **oscillatory vs static (fold change)** | **3 out of 3 experiments** |
| --- | --- | --- | --- | --- |
| Neo1 * | 12.19276753 | yes | 6.223807873 | no |
| ADAMTS1 * | 6.146311565 | yes | 0.903482083 | no |
| ADAMTS6 * | 5.384480005 | yes | 2.476399069 | yes |
| STC1 * | 0.179198509 | yes | 0.061512955 | yes |
| CD24 * | *not expr.* | | *not expr.* | |
| CAV1 * | 1.167182203 | yes | 0.9245087 | no |
| FGL2 * | *not expr.* | | *not expr.* | |
| ARL15 * | 3.283732847 | yes | 1.770079621 | yes |
| HS6ST3 * | *not expr.* | | *not expr.* | |
| RCAN1 * | 1.121556997 | no | 0.972940478 | no |
| GJA1 | 0.310167772 | yes | 1.157135349 | yes |
| ITGA9 | 1.401528105 | yes | 0.681870915 | yes |
| ALCAM | 1.652713615 | yes | 1.831181251 | yes |
| CD9 | 1.498924325 | yes | 1.157179075 | no |
| ACKR3 | 0.267824236 | yes | 0.238416694 | yes |
|  |  |  |  |  |
| **subcluster 2 (lower valve)** | **laminar vs static (fold change)** | **3 out of 3 experiments** | **Ocillatory vs static (fold change)** | **3 out of 3 experiments** |
| SCG3 * | 0.278142827 | yes | 0.629027694 | yes |
| HGF * | 0.251964712 | yes | 0.164124704 | yes |
| HBA2 * | *not expr.* | | *not expr.* | |
| ADM * | 0.191040991 | yes | 1.486181736 | no |
| GJA4 * | 2.651044936 | yes | 2.248904814 | yes |
| APOD * | 0.317189528 | yes | 1.232980615 | no |
| CFH * | 0.748795979 | no | 0.994669013 | no |
| FST * | 0.15558019 | yes | 3.311498712 | yes |
| SV2C * | *not expr.* | | *not expr.* | |
| CCSER1 * | *not expr.* | | *not expr.* | |
| CLU | 0.514120694 | yes | 3.943293373 | yes |
| ANGPT2 | 0.103917873 | yes | 0.640163172 | yes |
| CLDN11 | 0.56443795 | yes | 1.288967589 | no |
| FOXC2 | 0.6292129 | yes | 1.061346597 | no |

Summary of the flow-response patterns observed by bulk RNA sequencing in primary dermal LECs exposed to either laminar or oscillatory-like shear stress as compared to *in vitro* culture under static conditions. The flow-response patterns of selected marker genes characterizing LECs of the upper (subcluster 1) or lower (subcluster 2) valve leaflet (Fig. 5 B) are shown. Genes with an asterisk (*) represent the top 10 DEGs of the respective subclusters 1 and 2. Genes without asterisk correspond to further genes highlighted in Fig. 5 B. Genes for which the expected response pattern was observed in 3 out of 3 experiments performed are marked in green (expected upregulation) or red (expected downregulation), respectively. *Not expr*.: In 3 out of 3 experiments an FPKM (fragments per kilobase of transcript per million mapped reads) value of < 0.1 was measured for this gene.
