# Supplementary material for: Transcriptomics- and 3D imaging–based characterization of the lymphatic vasculature in human skin
Source: J Exp Med. 2025 Nov 4;223(1):e20242353. doi: 10.1084/jem.20242353 (PMC12584878; doi:10.1084/jem.20242353)
Supplement: Table S4 — lists antibodies. [file jem_20242353_tables4.docx]

**Table S4. Antibodies.** Combined list of antibodies used for immunofluorescence, flow cytometry, and hashtagging in scRNA-seq

| **Human whole-mount immunostaining** | | | |
| --- | --- | --- | --- |
| **Primary antibody** | **Clone** | **Company, catalog number** | **µg/mL*** |
| rat anti-human Podoplanin | NZ-1.2 | Sigma-Aldrich, MABC117 | 5.83 |
| goat anti-human LYVE-1 | - | R&D Systems, AF2089 | 2 |
| goat anti-human VE-cadherin | - | R&D Systems, AF938 | 0.2 |
| goat anti-human Neogenin 1 | - | R&D Systems, AF1079 | 10 |
| mouse anti-human CD24 | SN3 | Santa Cruz Biotech, sc-19585 | 2 |
| mouse anti-human CD24-FITC | SN3 | Santa Cruz Biotech, sc-19585 FITC | 2 |
| mouse anti-human alpha Smooth Muscle Actin | 1A4 | Thermofisher, 14-9760-80 | 1.67 |
| Mouse anti-human alpha Smooth Muscle Actin-Cy3 | 1A4 | Sigma-Aldrich, C6198 | 14 |
| rabbit anti-human von Willebrand Factor | - | DakoCytomation, A0082 | 5.6 |
| mouse anti-human FAK | 2C5B9 | Proteintech, 66258-1-Ig | 14.27 |
| goat anti-human CCL21 | - | R&D Systems, AF366 | 2 |
| rabbit anti-human CCL21 | - | Peptrotech, 500-P109 | 2 |
| mouse anti-human Foxc2 | G7 | Santa Cruz Biotech, sc-515234 | 4 |
| goat anti-human Prox1 | - | R&D Systems, AF2727 | 4 |
| rabbit anti-human Prox1 | - | ReliaTech, 102-PA32 | 4 |
| sheep anti-human Foxc2 | - | R&D Systems, AF5044 | 10 |
|  | | | |
| **Secondary antibody** | **Clone** | **Company, catalog number** | **µg/mL*** |
| donkey anti-rat IgG-AF405 | - | Thermo Scientific, A48268 | 6.6 |
| donkey anti-rat IgG-AF488 | - | Invitrogen, A-21208 | 6.6 |
| donkey anti-rat IgG-AF594 | - | Invitrogen, A-21209 | 6.6 |
| donkey anti-rat IgG-AF647 | - | Invitrogen, A78947 | 6.6 |
| donkey anti-mouse IgG-AF488 | - | Invitrogen, A-21202 | 6.6 |
| donkey anti-mouse IgG-AF594 | - | Invitrogen, A-21203 | 6.6 |
| donkey anti-mouse IgG-AF647 | - | Thermo Fisher Scientific, A-32787 | 6.6 |
| donkey anti-goat IgG-AF488 | - | Invitrogen, A-11055 | 6.6 |
| donkey anti-goat IgG-AF546 | - | Thermo Scientific, A-11056 | 6.6 |
| donkey anti-goat IgG-AF647 | - | Invitrogen, A-21447 | 6.6 |
| donkey anti-rabbit IgG-AF488 | - | Invitrogen, A-21206 | 6.6 |
| donkey anti-rabbit IgG-AF647 | - | Thermo Fisher Scientific, A31573 | 6.6 |
| donkey anti-sheep IgG-AF594 | - | Thermo Fisher Scientific, A11016 | 6.6 |
|  | | | |
| **Human sections immunofluorescence** | | | |
| **Primary antibody** | **Clone** | **Company, catalog number** | **µg/mL*** |
| mouse anti-human alpha smooth Muscle Actin | 1A4 | Thermofisher, 14-9760-80 | 0.5 |
| goat anti-human LYVE -1 | - | R&D Systems, AF2089 | 0.4 |
| rat anti-human Podoplanin | NZ-1.2 | Sigma-Aldrich, MABC117 | 3.5 |
| rabbit anti-human von Willebrand Factor | - | DakoCytomation, A0082 | 4 |
| **Secondary antibody** | **Clone** | **Company, catalog number** | **µg/mL*** |
| donkey anti-mouse IgG-AF488 | - | Invitrogen, A-21202 | 6.6 |
| donkey anti-goat IgG-AF546 | - | Thermo Scientific, A-11056 | 6.6 |
| donkey anti-rat IgG-AF594 | - | Invitrogen, A-21209 | 6.6 |
| donkey anti-rabbit IgG-AF647 | - | Thermo Fisher Scientific, A31573 | 6.6 |
|  | | | |
| **Human biopsy punches staining for light-sheet microscopy** | | | |
| **Primary antibody** | **Clone** | **Company, catalog number** | **µg/mL*** |
| rat anti-human Podoplanin | NZ-1.2 | Sigma-Aldrich, MABC117 | 17.5 |
| goat anti-human LYVE -1 | - | R&D Systems, AF2089 | 2 |
| rabbit anti-human LYVE -1 | - | ReliaTech, 102-PA50AG | 2 |
| goat anti-human Prox1 | - | R&D Systems, AF2727 | 2 |
| rabbit anti-human Prox1 | - | ReliaTech, 102-PA32 | 2 |
| goat anti-human VE-cadherin | - | R&D Systems, AF938 | 2 |
| mouse anti-human alpha smooth Muscle Actin | 1A4 | Thermofisher, 14-9760-80 | 5 |
| Rabbit anti-human Von Willebrand Factor | - | DakoCytomation, A0082 | 32 |
| **Secondary antibody** | **Clone** | **Company, catalog number** | **µg/mL*** |
| donkey anti-rat IgG-AF488 | - | Invitrogen, A-21208 | 6.6 |
| donkey anti-rat IgG-AF594 | - | Invitrogen, A-21209 | 6.6 |
| donkey anti-rat IgG-AF647 | - | Invitrogen, A78947 | 6.6 |
| donkey anti-goat IgG-AF546 | - | Thermo Scientific, A-11056 | 6.6 |
| donkey anti-goat IgG-AF594 | - | Invitrogen, A-11058 | 6.6 |
| donkey anti-goat IgG-AF647 | - | Invitrogen, A-21447 | 6.6 |
| donkey anti-mouse IgG-AF488 | - | Invitrogen, A-21202 | 6.6 |
| donkey anti-mouse IgG-AF594 | - | Invitrogen, A-21203 | 6.6 |
| donkey anti-mouse IgG-AF594 | - | Invitrogen, A-21203 | 6.6 |
| donkey anti-rabbit IgG-AF647 | - | Thermo Fisher Scientific, A31573 | 6.6 |
|  | | | |
| **Human flow cytometry** | | | |
| **Primary antibody** | **Clone** | **Company, catalog number** | **µg/mL*** |
| Human TruStain FcX (Fc receptor blocking) | - | Biolegend, 422301 | 5 µl per 100 µl |
| mouse anti-human CD45-BV421 | HI30 | Biolegend, 304032 | 0.25 |
| mouse anti-human CD31-PE | WM59 | Biolegend, 303106 | 0.1 |
| rat anti-human podoplanin-PE/Cy7 | NC-08 | Biolegend, 337014 | 0.1 |
| goat anti-human LYVE -1 | - | R&D Systems, AF2089 | 4 |
| mouse anti-human CD24-FITC | SN3 | Santa Cruz Biotech, sc-19585 FITC | 2 |
| **Secondary** | **Clone** | **Company, catalog number** | **µg/mL*** |
| donkey anti-goat IgG-AF647 | - | Invitrogen, A-21447 | 6.6 |
|  | | | |
| **Mouse whole-mount immunofluorescence** | | | |
| **Primary antibody** | **Clone** | **Company, catalog number** | **µg/mL*** |
| rat anti-mouse CD24-AF647 | M1/69 | Biolegend, 101818 | 2.5 |
| rat IgG2b,k-AF647 | - | Biolegend, 400626 | 2.5 |
| rat anti-mouse CD31-PE | MEC13.3 | Biolegend, 102508 | 1 |
| rat anti-mouse CD31 | MEC13.3 | BD Pharmingen, 53370 | 1 |
| rat anti-mouse Podocalyxin | 192703 | R&D systems, MAB1556 | 0.1 |
| rabbit anti-mouse Laminin α5 | 4G6 | Kind gift from Lydia Sorokin (Ringelmann et al., 1999) | 1:1000 from serum |
| goat anti-mouse Vegfr3 | - | R&D systems, AF743 | 1 |
| mouse anti-human alpha Smooth Muscle Actin-Cy3 | 1A4 | Sigma, C6198 | 1 |
| goat anti-mouse Vegfr2 | - | R&D systems, AF644 | 2 |
| Goat anti-mouse Integrin alpha 9 | - | R&D systems, AF3827 | 2 |
| **Secondary antibody** | **Clone** | **Company, catalog number** | **µg/mL*** |
| donkey anti-rat IgG-AF488 | - | Life Technologies, A21208 | 4 |
| donkey anti-rat IgG-AF405 | - | Thermo Scientific, A48268 | 6.6 |
| donkey anti-rabbit-AF555 | - | Life Technologies, A31572 | 4 |
| donkey anti-goat-AF647 | - | Life Technologies, A21447 | 4 |
|  | | | |
| **Mouse flow cytometry** | | | |
| **Primary antibody** | **Clone** | **Company, catalog number** | **µg/mL*** |
| rat anti-mouse CD16/32 (Fc receptor blocking) | - | Biolegend, 101302 | 50 |
| syrian hamster anti-mouse Podoplanin-PE/Cy7 | 8.1.1 | Biolegend, 127412 | 1 |
| rat anti-mouse CD31-BV421 | MEC13.3 | BD Biosciences, 562939 | 1 |
| rat anti-mouse CD24-AF647 | M1/69 | Biolegend, 101818 | 2.5 |
| ** final antibody concentrations used in µg/mL* | | | |

| **Anti-human hashtag antibodies used for scRNAseq** | | | | | |
| --- | --- | --- | --- | --- | --- |
| **Sequencing run** | **Donor number and tissue type** | **Hashtag antibody number** | **Oligonucleotide sequence of the hashtag antibody** | **Biolegend catalog number** | **TotalSeq** |
| **1** | 1-skin | 6 | GGTTGCCAGATGTCA | 394641 | B |
|  | 1-adipose | 7 | TGTCTTTCCTGCCAG | 394643 | B |
|  | 2-adipose | 8 | CTCCTCTGCAATTAC | 394675 | B |
| **2** | 3-skin | 6 | GGTTGCCAGATGTCA | 394641 | B |
|  | 3-adipose | 7 | TGTCTTTCCTGCCAG | 394643 | B |
|  | 4-skin | 8 | CTCCTCTGCAATTAC | 394675 | B |
|  | 4-adipose | 9 | CAGTAGTCACGGTCA | 394647 | B |
| **3** | 5-skin | 4 | AGTAAGTTCAGCGTA | 394607 | A* |
|  | 5-adipose | 5 | AAGTATCGTTTCGCA | 394609 | A* |
|  | 6-skin | 6 | GGTTGCCAGATGTCA | 394641 | B |
|  | 6-adipose | 7 | TGTCTTTCCTGCCAG | 394643 | B |
|  | 7-skin | 8 | CTCCTCTGCAATTAC | 394675 | B |
|  | 7-adipose | 9 | CAGTAGTCACGGTCA | 394647 | B |
| ** By mistake, TotalSeq-A instead of TotalSeq-B Hashtag antibodies were used for both the skin and adipose tissue samples from donor 5. Consequently, LECs from donor 5 could not be assigned to their specific tissue origin (skin or adipose tissue) and were instead recovered by selecting the unhashed barcodes*. | | | | | |

**References**

Ringelmann, B., C. Roder, R. Hallmann, M. Maley, M. Davies, M. Grounds, and L. Sorokin. 1999. Expression of laminin alpha1, alpha2, alpha4, and alpha5 chains, fibronectin, and tenascin-C in skeletal muscle of dystrophic 129ReJ dy/dy mice. *Exp. Cell Res.* 246:165-182. https://doi.org/10.1006/excr.1998.4244
